# SO_2_F_2_ mediated cascade dehydrogenative Morita–Baylis–Hillman reaction of the C(sp^3^)–H of primary alcohols with the C(sp^2^)–H of electron-deficient olefins for the assembly of allylic alcohols†

**DOI:** 10.1039/c9ra05346h

**Published:** 2019-09-20

**Authors:** Ying Jiang, Njud S. Alharbi, Bing Sun, Hua-Li Qin

**Affiliations:** School of Chemistry, Chemical Engineering and Life Sciences, Wuhan University of Technology Wuhan 430070 PR China bing.sun@whut.edu.cn qinhuali@whut.edu.cn; Biotechnology Research Group, Department of Biological Sciences, Faculty of Science, King Abdulaziz University Jeddah Saudi Arabia njud_alharbi@yahoo.com

## Abstract

A cascade dehydrogenative Morita–Baylis–Hillman reaction of the C(sp^3^)–H of primary alcohols with the C(sp^2^)–H of electron-deficient olefins for forming allylic alcohols mediated by SO_2_F_2_ was developed. This method provides a mild process for the preparation of allylic alcohol moieties without the requirement of transition metals.

## Abstract 2

A cascade dehydrogenative Morita–Baylis–Hillman reaction of the C(sp^3^)–H of primary alcohols with the C(sp^2^)–H of electron-deficient olefins for forming allylic alcohols mediated by SO_2_F_2_ was developed.

## Graphical abstract

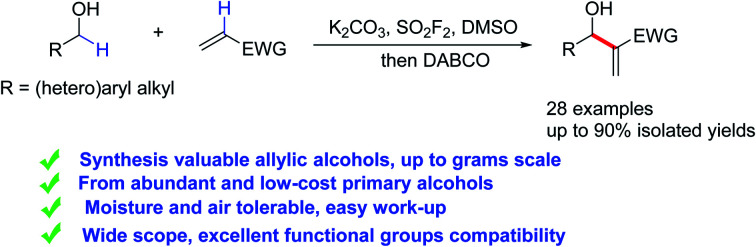

Allylic alcohols are valuable scaffolds that are used in the construction of multifunctional building blocks and complex natural products.^1^ The versatility of these molecules has been demonstrated in the preparation of a series of biologically active compounds.^2^ A representative protocol for the synthesis of allylic alcohols is the well-known Morita–Baylis–Hillman reaction, one of the most widely applied methods for C–C bond formation.^3^

In fact, C–C bond formation is among the most significant processes in chemistry and plays a central role in the construction of new organic molecules,^4^ in which transition metal catalysed C–C bond formation has particularly attracted great interest in recent years.^5^ For instance, a reaction for the direct formation of C–C bonds using two different unfunctionalized C–H bond partners was reported (Scheme 1a).^6^ Despite the great advantages of these dehydrogenative reactions for the formation of C–C bonds^7^ there are still certain limitations, such as that precious metal catalysts are still required (metal catalysts are sometimes undesirable).^8^ To overcome the limitations, we developed a new protocol for the formation of allylic alcohol motifs using abundant and inexpensive reagents.

**Scheme 1:**
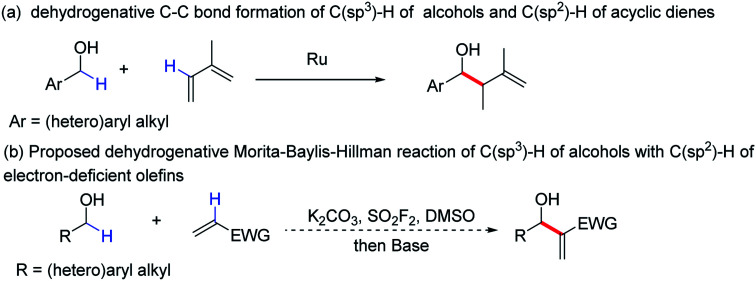
Dehydrogenative reactions for the formation of C–C bonds.

Alcohols, as a class of cheap and abundant industrial chemicals, have great advantages in green chemistry and organic synthesis.^9^ Sulfuryl fluoride (SO_2_F_2_), is also another inexpensive (about $1 per kg) and abundant chemical, which has attracted significant attention for chemical transformation.^10^ As part of our continuous efforts on the use of SO_2_F_2_ in exploring new synthetic methods to access important chemicals,^10*c*–*l*^ herein, we report a one-pot process for the construction of valuable allylic alcohols through a dehydrogenative Morita–Baylis–Hillman reaction of the C(sp^3^)–H of primary alcohols with the C(sp^2^)–H of electron-deficient olefins (Scheme 1b).

Initially, we examined the feasibility of this transformation using (4-nitrophenyl)methanol 1a as a model substrate to react with methyl methacrylate 2a to generate the corresponding allylic alcohol 3a. It has been widely established that tertiary amines such as trimethylamine (Me_3_N), 1,8-diazabicyclo[5.4.0]undec-7-ene (DBU) and 1,4-diazabicyclo[2.2.2]octane (DABCO) are effective for the Baylis–Hillman reaction,^11^ which inspired us to carry out our preliminary experiments using these bases. The use of Me_3_N and DBU provided the desired product 3a in only 5% and 41% yields, respectively (Table 1, entries 1 and 2). Excitingly, when 3.0 equivalents of DABCO was used, the yield of product 3a increased to 74% (Table 1, entry 3), indicating that DABCO is a suitable base for the desired transformation. In order to maximize the yield of allylic alcohol, the temperature was elevated to 40 °C and an obvious increase in the yield was achieved (Table 1, entry 4, 83% yield). Further elevating the temperature to 50 °C resulted in a slightly lower yield of 3a (Table 1, entry 5). The loading of DABCO was subsequently examined (Table 1, entry 6 and 7), which revealed that a 3.0-fold excess was an appropriate amount. Decreasing the amount of methyl acrylate 2a to 1.0 equivalent resulted in a significant decrease in the formation of 3a (Table 1, 52% *vs.* 83%; entry 8 *vs.* entry 4). When 5.0 equivalents of methyl acrylate 2a was used, the yield of product 3a was not obviously increased compared with the use of 3.0 equivalents of acrylate 2a (Table 1, 84% *vs.* 83%; entry 9 *vs.* entry 4). Therefore, the parameters in entry 4 of Table 1 were selected as the standard conditions for further substrate scope examination.

**Table 1:** Optimization of the reaction conditions^a^

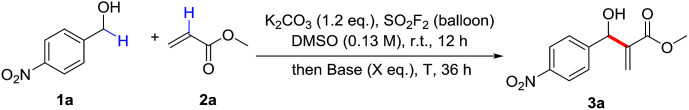				
Entry	Base (*X* eq.)	2a (*Y* eq.)	Temperature (°C)	Yield^b^ (3a, %)
1	Me_3_N (3.0)	3.0	rt	5
2	DBU (3.0)	3.0	rt	41
3	DABCO (3.0)	3.0	rt	74
**4**	**DABCO (3.0)**	**3.0**	**40**	**83**
5	DABCO (3.0)	3.0	50	77
6	DABCO (1.0)	3.0	40	42
7	DABCO (5.0)	3.0	40	85
8	DABCO (3.0)	1.0	40	52
9	DABCO (3.0)	5.0	40	84

a General reaction conditions: a mixture of (4-nitrophenyl)methanol (1a, 0.2 mmol), K_2_CO_3_ (0.24 mmol, 1.2 eq.) and DMSO (1.5 mL, 0.13 M) under an atmosphere of SO_2_F_2_ (balloon) was stirred at room temperature for 12 h before base (*X* eq.) and methyl acrylate 2a (*Y* eq.) were added. The resulting mixture was stirred at the corresponding temperature for an additional 36 h.

b HPLC yields using the pure methyl 2-(hydroxy(4-nitrophenyl)methyl)acrylate (3a, 0.2 mmol) as the external standard (*t*_R_ = 3.021 min, *λ*_max_ = 272.5 nm, MeOH/H_2_O = 70 : 30 (v/v)).

We subsequently investigated the substrate scope of benzyl alcohols 1 under the optimized conditions, and the results are summarized in Table 2. Not surprisingly, a variety of substituted benzyl alcohols 1 smoothly reacted with methyl acrylate 2a to provide their corresponding allylic alcohols (3a–3n) in moderate to excellent yields. Both electron-withdrawing (1a–1j) and electron-donating (1l and 1m) groups on the aryl rings were all well tolerated under the standard reaction conditions, even though the reactivity of electron-deficient substrates was somewhat superior to those of electron-rich ones. Notably, the positions of substituents on the aryl rings did seem to have much influence on the efficiency. Sterically hindered *ortho*-position substituted benzylic alcohols were used to furnish the desired products in slightly lower yields than the *para*-position functionalized substrates (*e.g.*3a*vs.*3b). It was noteworthy to find that a broad range of heterocyclic benzylic alcohols bearing sulfur or nitrogen atoms on the aryl rings (1o–1u) were smoothly converted to their corresponding allylic alcohols in moderate yields. Remarkably, this method is compatible with various electron-deficient terminal alkenes (2v, 2w and 2x) for the formation of their corresponding allylic alcohol products (3v, 3w and 3x).

**Table 2:** Substrate scope examination of the dehydrogenative Morita–Baylis–Hillman reaction^a^

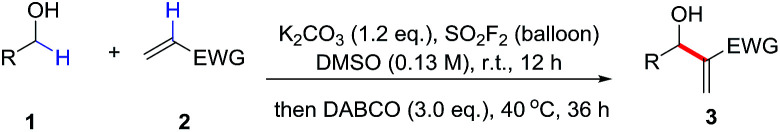
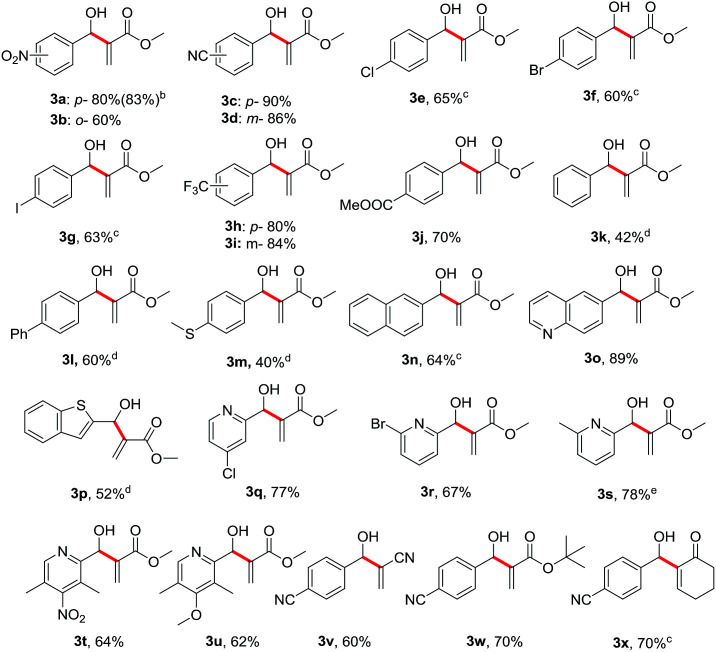

a General reaction conditions: benzyl alcohols (1, 1.0 mmol), K_2_CO_3_ (1.2 mmol, 1.2 eq.), DMSO (7.5 mL, 0.13 M), SO_2_F_2_ balloon, rt, 12 h. Then DABCO (3.0 mmol, 3.0 eq.) and methyl acrylate 2a (3.0 mmol, 3.0 eq.), 40 °C, 36 h. Isolated yield.

b HPLC yield.

c 40 °C, 72 h.

d 40 °C, 5 days.

e 40 °C, 6 h.

To further demonstrate the generality and substrate scopes of the process, we also examined some long-chain aliphatic alcohols under the standard conditions for the coupling with methyl acrylate 2a (Table 3). Not surprisingly, these aliphatic alcohols (1y–1ab) were also smoothly converted to their corresponding allylic alcohols (3y–3ab) in moderate yields.

**Table 3:** Dehydrogenative Morita–Baylis–Hillman reaction of long-chain aliphatic alcohols with methyl acrylate 2a^a^

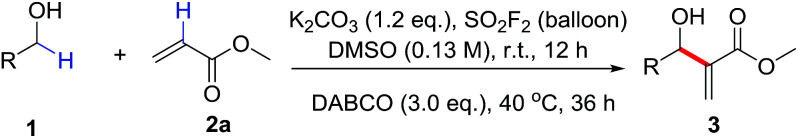
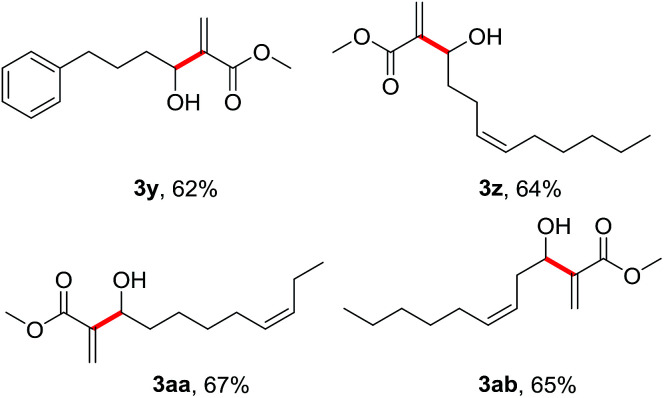

a General reaction conditions: long-chain aliphatic alcohols (1, 1.0 mmol), K_2_CO_3_ (1.2 mmol, 1.2 eq.), DMSO (7.5 mL, 0.13 M), SO_2_F_2_ balloon, rt, 12 h. Then DABCO (3.0 mmol, 3.0 eq.) and methyl acrylate 2a (3.0 mmol, 3.0 eq.), 40 °C, 36 h. Isolated yield.

In order to demonstrate the practicality of the process, a couple of gram-scale reactions were performed under the standard conditions (Scheme 2). Despite a slight decline in the yield, 1i was successfully converted to its corresponding allylic alcohol 3i in 70% yield. Pyridine-containing benzylic alcohol 1s was also smoothly transformed into the corresponding allylic alcohol 3s in 75% yield without any deterioration in efficiency.

**Scheme 2:**
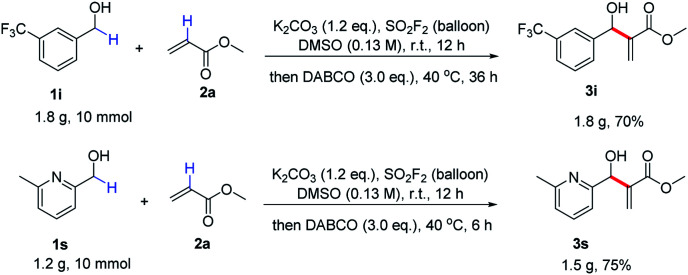
Gram-scale reactions.

In conclusion, we have developed a cascade dehydrogenative Morita–Baylis–Hillman reaction of the C(sp^3^)–H of primary alcohols with the C(sp^2^)–H of electron-deficient olefins to form allylic alcohols mediated by SO_2_F_2_. This new protocol has the features of a wide scope and great functional group compatibility. Using this method, inexpensive, easily accessible, and abundant alcohols can be directly transformed to highly valuable allylic alcohols under mild conditions without the use of precious transition metals.

## Conflicts of interest

There are no conflicts to declare.

## Supplementary Material

RA-009-C9RA05346H-s001
