# Supplementary material for: SO2F2 mediated cascade dehydrogenative Morita–Baylis–Hillman reaction of the C(sp3)–H of primary alcohols with the C(sp2)–H of electron-deficient olefins for the assembly of allylic alcohols
Source: RSC Adv. 2019 Sep 20;9(51):29784–7. doi: 10.1039/c9ra05346h (PMC9072135; doi:10.1039/c9ra05346h)
Supplement: RA-009-C9RA05346H-s001 [file RA-009-C9RA05346H-s001.pdf]

Electronic Supplementary Material (ESI) for RSC Advances.
This journal is © The Royal Society of Chemistry 2019

Supporting Information

SO_2F_2 mediated cascade dehydrogenative Morita-Baylis-Hillman reaction of C(sp^3)-H of primary alcohols with C(sp^2)-H of electron-deficient olefins for assembly of allylic alcohols

Ying Jiang[a] Njud S. Alharbi[b] Bing Sun,[a*] and Hua-Li Qin,[a*]

[a]School of Chemistry, Chemical Engineering and Life Science, Wuhan University of Technology, Wuhan, Hubei Province 430070, People's Republic of China

[b]Biotechnology Research group, Deportment of Biological Sciences, Faculty of Science, King Abdulaziz University, Jeddah, Saudi Arabia

Table of Content

1. General Consideration………………………………………………………………… S2

2. Screening the optimized reaction conditions ………………………………………… S3

3. General procedure………………..……………………………………………………… .S5

4. Product Characterization……………………………………………………………….S6

5. Reference…………………………………………………………………………………….S14

6. NMR Spectra…………………………………………………………………………...S15

1. General considerations

All reactions were carried out in dried glassware. All reagents were purchased from commercial sources and used without further purification. Unless otherwise specified, NMR spectra were recorded in $CDCl_3$ or DMSO-d_6 on a 500 or 400 MHz (for ^1H), 471 or 376 MHz (for ^{19}F), 126 MHz (for ^{13}C) spectrometer. All chemical shifts were reported in ppm relative to TMS (^1H NMR, 0 ppm) as internal standards. The HPLC experiments were carried out on a Waters e2695 instrument (column: J&K, RP-C18, 5 μm, 4.6 × 150 mm), and the yields of the products were determined by using the corresponding pure compounds as the external standards. The coupling constants were reported in Hertz (Hz). The following abbreviations were used to explain the multiplicities: s = singlet, d = doublet, t = triplet, q = quartet, m = multiplet, br = broad. MS experiments were performed on a TOF-Q ESI or CI/EI instrument. IR experiments were performed on a Nicolet6700 instrument. Melting points were measured and uncorrected.

2. Screening the optimized reaction conditions

Table 1 Screening the Base[a]

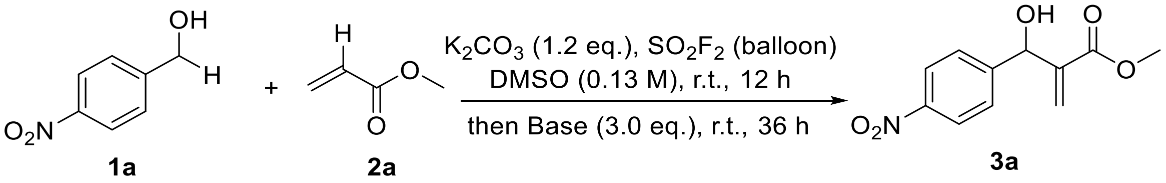

Entry	Base (3.0 eq.)	Yield (**3a**, %)[b]
1	$(Me)_3N$	5
2	DBU	41
**3**	**DABCO**	**74**

[a]General condition: A mixture of (4-nitrophenyl)methanol (**1a**, 0.2 mmol), K_2CO_3 (0.24 mmol, 1.2 eq.) and DMSO (1.5 mL, 0.13 M) under an atmosphere of SO_2F_2 (balloon) was stirred at room temperature for 12 hours before methyl acrylate **2a** (0.6 mmol, 3.0 eq.), Base (3.0 eq.) were added and then the mixture was stirred at room temperature for an additional 36 hours [b]HPLC yields using the pure methyl 2-(hydroxy(4-nitrophenyl)methyl)acrylate (**3a**, 0.2 mmol) as the external standard (t_R = 3.021min, λ_{max} = 272.5 nm, MeOH/H_2O = 70 : 30 (v / v)).

Table 2 Screening the temperature[a]

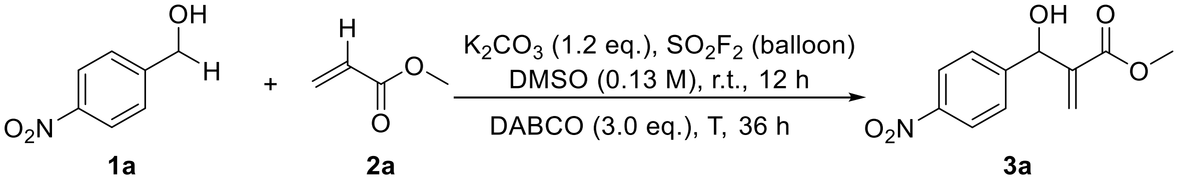

Entry	T (°C)	Yield (**3a**, %)[b]
1	rt	74
**2**	**40**	**83**
3	50	77
4	60	71

[a]General reaction condition: A mixture of (4-nitrophenyl)methanol (**1a**, 0.2 mmol), K_2CO_3 (0.24 mmol, 1.2 eq.) and DMSO (1.5 mL, 0.13 M) under an atmosphere of SO_2F_2 (balloon) was stirred at room temperature for 12 hours before methyl acrylate **2a**

(0.6 mmol, 3.0 eq.), DABCO (0.6 mmol, 3.0 eq.) were added and then the mixture was stirred at corresponding temperature for an additional 36 hours [b]HPLC yields using the pure methyl 2-(hydroxy(4-nitrophenyl)methyl)acrylate (**3a**, 0.2 mmol) as the external standard (t_R = 3.021 min, λ_{max} = 272.5 nm, MeOH/H_2O = 70 : 30 (v / v)).

Table 3 Screening the loading amount of DABCO[a]

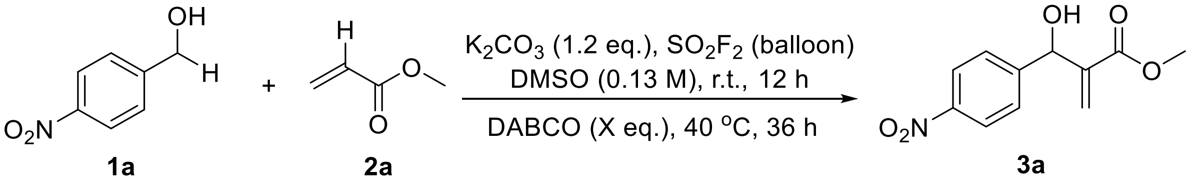

Entry	DABCO Loading (X eq.)	Yield (**3a**, %)[b]
1	1	42
2	2	73
**3**	**3**	**83**
4	4	82
5	5	85

[a]General reaction condition: A mixture of (4-nitrophenyl)methanol (**1a**, 0.2 mmol), K_2CO_3 (0.24 mmol, 1.2 eq.) and DMSO (1.5 mL, 0.13 M) under an atmosphere of SO_2F_2 (balloon) was stirred at room temperature for 12 hours before methyl acrylate **2a** (0.6 mmol, 3.0 eq.), DABCO (X eq.) were added and then the mixture was stirred at 40 °C for an additional 36 hours [b]HPLC yields using the pure methyl 2-(hydroxy(4-nitrophenyl)methyl)acrylate (**3a**, 0.2 mmol) as the external standard (t_R = 3.021 min, λ_{max} = 272.5 nm, MeOH/H_2O = 70 : 30 (v / v)).

Table 4 Screening Methyl acrylate Loading[a]

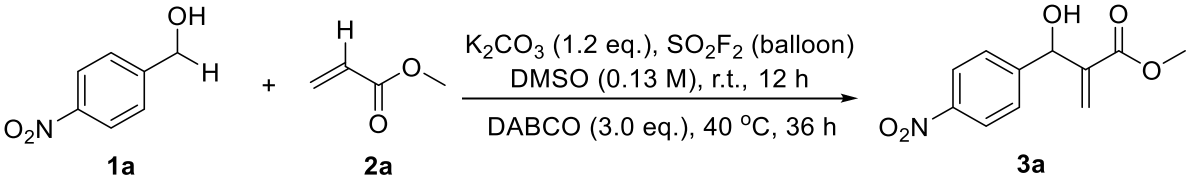

Entry	**2a** Loading (X eq.)	Yield (**3a**, %)[b]
1	1	52

2	2	62
**3**	**3**	**83**
4	4	84
5	5	84

[a]General reaction condition: A mixture of (4-nitrophenyl)methanol (**1a**, 0.2 mmol), K_2CO_3 (0.24 mmol, 1.2 eq.) and DMSO (1.5 mL, 0.13 M) under an atmosphere of SO_2F_2 (balloon) was stirred at room temperature for 12 hours before methyl acrylate **2a** (X eq.), DABCO (0.6 mmol, 3.0 eq.) were added and then the mixture was stirred at 40 °C for an additional 36 hours [b]HPLC yields using the pure methyl 2-(hydroxy(4-nitrophenyl)methyl)acrylate (**3a**, 0.2 mmol) as the external standard (t_R = 3.021 min, λ_{max} = 272.5 nm, MeOH/H_2O = 70 : 30 (v / v))..

3. General procedure

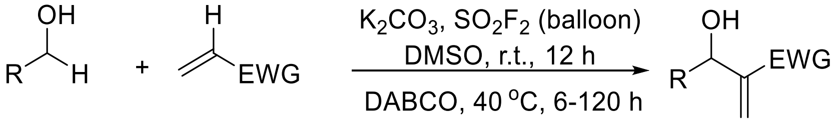

1 **2** **3**

Alcohol **1** (1.0 mmol, 1.0 eq.), K_2CO_3 (165.9 mg, 1.2 mmol, 1.2 eq.), and DMSO (7.5 mL) added to an oven-dried reaction tube (20 ml) that was equipped with a stirrer bar. The tube was fitted with a plastic stopper and SO_2F_2 gas was introduced into the stirring reaction mixture by bubbling from an SO_2F_2 balloon at room temperature for 12 h. After the alcohol was completely consumed (by TLC), **2** (3.0 mmol, 3.0 eq.), DABCO (3.0 mmol, 3.0 eq.) were added into the reaction mixture, and reacted at 40 °C for 6-120 hours. After that, the reaction diluted with water and extracted with EtOAc (3×20 ml) and the combined organic layer was washed with brine, dried over anhydrous Na_2SO_4, and concentrated to dryness. The residue was purified by column chromatography on silica gel to afford the desired **3**.

4. Product Characterization

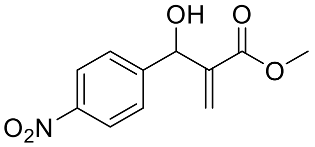

3a

Methyl 2-(hydroxy(4-nitrophenyl)methyl)acrylate (**3a**). White solid (190 mg from **1a**, isolated yield 80%). HPLC yield 83% (using methyl 2-(hydroxy(4-nitrophenyl)methyl)acrylate (**3a**) (t_R = 3.021 min, λ_{max} = 272.5 nm, MeOH/H_2O = 70 : 30 (v / v))as the external standard). The NMR data is identical to that reported in literature.[1] ^{1}H NMR ($CDCl_3$, 500 MHz) δ 8.18 (d, *J* = 8.7 Hz, 2H), 7.56 (d, *J* = 8.5 Hz, 2H), 6.38 (s, 1H), 5.88 (s, 1H), 5.62 (d, *J* = 6.0 Hz, 1H), 3.73 (s, 3H), 3.44 (d, *J* = 6.1 Hz, 1H)

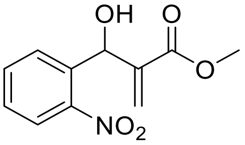

3b

Methyl 2-(hydroxy(2-nitrophenyl)methyl)acrylate (**3b**). Yellow oil (142 mg from **1b**, isolated yield 60%). The NMR data is identical to that reported in literature.[2] ^{1}H NMR ($CDCl_3$, 500 MHz) δ 7.93(d, *J* = 8.2 Hz, 1H), 7.74 (d, *J* = 7.8 Hz, 1H), 7.63 (t, *J* = 7.5 Hz, 1H), 7.45 (t, *J* = 7.6 Hz, 1H), 6.34 (s, 1H), 6.19 (s, 1H), 5.71 (s, 1H), 3.71 (s, 3H).

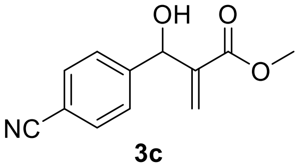

3c

Methyl 2-((4-cyanophenyl)(hydroxy)methyl)acrylate (**3c**). Colorless oil (195 mg from **1c**, isolated yield 90%). The NMR data is identical to that reported in literature.[3] ^{1}H NMR ($CDCl_3$, 500 MHz) δ 7.60 (d, *J* = 8.1 Hz, 2H), 7.48 (d, *J* = 8.1 Hz, 2H), 6.35 (s, 1H), 5.87 (s, 1H), 5.56 (d, *J* = 4.6 Hz, 1H), 3.70 (s, 3H), 3.59 (d, *J* = 5.3 Hz, 1H).

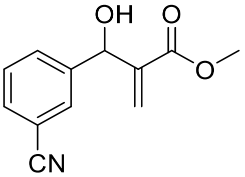

3d

Methyl 2-((3-cyanophenyl)(hydroxy)methyl)acrylate (**3d**). Colorless oil (187mg from **1d**, isolated yield 86%). The NMR data is identical to that reported in literature.[4] ^{1}H NMR ($CDCl_3$, 500 MHz) δ 7.67 (s, 1H), 7.62 (d, *J* = 7.8 Hz, 1H), 7.55 (d, *J* = 7.6 Hz, 1H), 7.44 (t, *J* = 7.7 Hz, 1H), 6.37 (s, 1H), 5.88 (s, 1H), 5.55 (d, *J* = 4.5 Hz, 1H), 3.72

(s, 3H), 3.50 (d, *J* = 5.4 Hz, 1H).

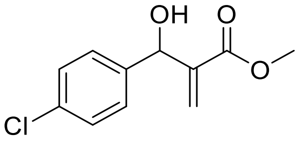

3e

Methyl 2-((4-chlorophenyl)(hydroxy)methyl)acrylate (**3e**). Colorless oil (147 mg from **1e**, isolated yield 65%). The NMR data is identical to that reported in literature.[4] ^{1}H NMR ($CDCl_3$, 500 MHz) δ 7.30 (s, 4H), 6.33 (s, 1H), 5.83 (s, 1H), 5.51 (s, 1H), 3.71 (s, 3H), 3.26 (brs, 1H).

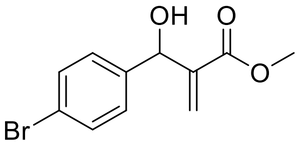

3f

Methyl 2-((4-bromophenyl)(hydroxy)methyl)acrylate (**3f**). Light yellow oil (163 mg from **1f**, isolated yield 60%). The NMR data is identical to that reported in literature.[4] ^{1}H NMR ($CDCl_3$, 500 MHz) δ 7.43 (d, *J* = 8.2 Hz 2H), 7.21 (d, *J* = 8.3 Hz, 2H), 6.31 (s, 1H), 5.83 (s, 1H), 5.47 (s, 1H), 3.69 (s, 3H), 3.45 (brs, 1H).

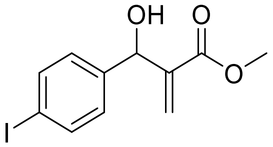

3g

Methyl 2-(hydroxy(4-iodophenyl)methyl)acrylate (**3g**). White solid (200 mg from **1g**, isolated yield 63%). M. p. 108~110 °C, ^{1}H NMR ($CDCl_3$, 500 MHz) δ 7.67 (d, *J* = 8.2 Hz, 2H), 7.12 (d, *J* = 8.2 Hz, 2H), 6.34 (s, 1H), 5.83 (s, 1H), 5.49 (d, *J* = 3.2 Hz, 1H), 3.73 (s, 3H), 3.14 (d, *J* = 5.0 Hz, 1H). ^{13}C NMR ($CDCl_3$, 126 MHz) δ 166.8, 141.7, 141.2, 137.7, 128.7, 126.6, 93.6, 73.0, 52.2. ESI-MS HRMS calculated for $C_{11}H_{12}IO_3$ $[M+H]^+$ 318.9831, found: 318.9837.

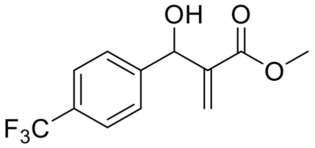

3h

Methyl 2-(hydroxy(4-(trifluoromethyl)phenyl)methyl)acrylate (**3h**). Colorless oil (208 mg from **1h**, isolated yield 80%). The NMR data is identical to that reported in literature.[4] ^{1}H NMR ($CDCl_3$, 500 MHz) δ 7.60 (d, *J* = 8.0 Hz, 2H), 7.50 (d, *J* = 8.0 Hz, 2H), 6.37 (s, 1H), 5.85 (s, 1H), 5.59 (d, *J* = 5.2 Hz, 1H), 3.73 (s, 3H), 3.36 (d, *J* = 5.8

Hz, 1H).

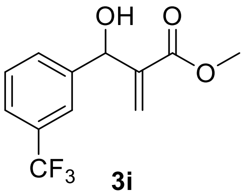

3i

Methyl 2-(hydroxy(3-(trifluoromethyl)phenyl)methyl)acrylate (**3i**). Light yellow oil (218 mg from **1i**, isolated yield 84%). The NMR data is identical to that reported in literature.[5] ^{1}H NMR ($CDCl_3$, 500 MHz) δ 7.64 (s, 1H), 7.54 (t, J = 7.6 Hz, 2H), 7.44 (t, J = 7.8 Hz, 1H), 6.36 (s, 1H), 5.85 (s, 1H), 5.58 (s, 1H), 3.71 (s, 3H), 3.49 (brs, 1H).

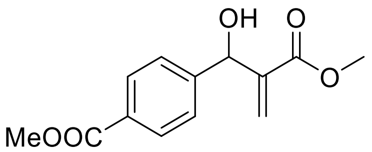

3j

Methyl 4-(1-hydroxy-2-(methoxycarbonyl)allyl)benzoate(**3j**). White solid (175 mg from **1j**, isolated yield 70%). The NMR data is identical to that reported in literature.[6] ^{1}H NMR ($CDCl_3$, 500 MHz) δ 8.00 (d, J = 8.2 Hz, 2H), 7.44 (d, J = 8.3 Hz, 2H), 6.35 (s, 1H), 5.83 (s, 1H), 5.59 (d, J = 5.2 Hz, 1H), 3.90 (s, 3H), 3.71 (s, 3H), 3.33 (d, J = 5.8 Hz, 1H).

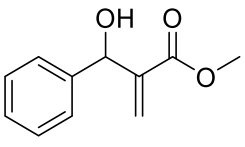

3k

Methyl 2-(hydroxy(phenyl)methyl)acrylate (**3k**). Light yellow oil (81 mg from **1k**, isolated yield 42%). The NMR data is identical to that reported in literature.[5] ^{1}H NMR ($CDCl_3$, 500 MHz) δ 7.38-7.33 (m, 4H), 7.28 (t, J = 7.1 Hz, 1H), 6.34 (s, 1H), 5.84 (s, 1H), 5.56 (s, 1H), 3.72 (s, 3H), 3.13 (brs, 1H).

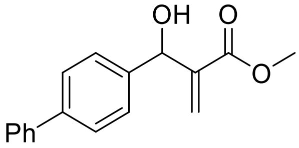

3l

Methyl 2-([1,1'-biphenyl]-4-yl(hydroxy)methyl)acrylate (**3l**). White solid (161 mg from **1l**, isolated yield 60%). The NMR data is identical to that reported in literature.[7] ^{1}H NMR ($CDCl_3$, 500 MHz) δ 7.60-7.58 (m, 4H) 7.47-7.43 (m, 4H), 7.35 (t, J = 7.3 Hz, 1H), 6.38 (s, 1H), 5.91 (s, 1H), 5.63 (d, J = 5.3 Hz, 1H), 3.75 (s, 3H), 3.08 (d, J = 5.5 Hz, 1H).

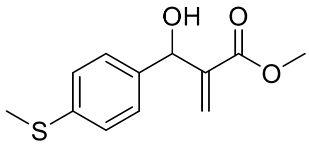

3m

Methyl 2-(hydroxy(4-(methylthio)phenyl)methyl)acrylate (**3m**). White solid (95 mg from **1m**, isolated yield 40%) The NMR data is identical to that reported in literature.[8] ^{1}H NMR ($CDCl_3$, 500 MHz) δ 7.29 (d, J = 8.3 Hz, 2H) 7.22 (d, J = 8.3 Hz, 2H), 6.33 (s, 1H), 5.85 (s, 1H), 5.52 (d, J = 4.3 Hz, 1H), 3.72 (s, 3H), 3.05 (d, J = 5.2 Hz, 1H), 2.47 (s, 3H).

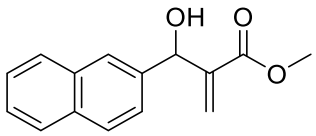

3n

Methyl 2-(hydroxy(naphthalen-2-yl)methyl)acrylate (**3n**). White solid (155 mg from **1n**, isolated yield 64%). The NMR data is identical to that reported in literature.[5] ^{1}H NMR ($CDCl_3$, 500 MHz) δ 7.86-7.82 (m, 4H) 7.50-7.46 (m, 3H), 6.38 (s, 1H), 5.89 (s, 1H), 5.74 (s,1H), 3.72 (s, 3H), 3.25 (brs, 1H).

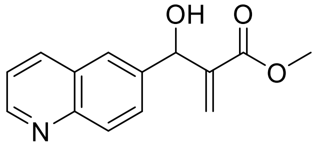

3o

Methyl 2-(hydroxy(quinolin-6-yl)methyl)acrylate (**3o**). White solid (217 mg from **1o**, isolated yield 89%). M.p. 119~120 °C. ^{1}H NMR ($CDCl_3$, 500 MHz) δ 8.79 (dd, J = 4.2 Hz, 1.6 Hz, 1H), 8.10 (d, J = 8.1 Hz, 1H), 8.00 (d, J = 8.9 Hz, 1H), 7.82 (s, 1H), 7.66 (dd, J = 8.6 Hz, 1.6 Hz, 1H), 7.35 (dd, J = 8.2 Hz, 4.2 Hz, 1H), 6.38 (s, 1H), 5.96 (s, 1H), 5.77 (s, 1H), 4.25 (brs, 1H), 3.69 (s, 3H). ^{13}C NMR ($CDCl_3$, 126 MHz) δ 166.8, 150.4, 147.8, 142.0, 140.0, 136.5, 129.5, 128.5, 128.1, 126.5, 125.5, 121.4, 72.8, 52.1. ESI-MS HRMS calculated for $C_{14}H_{14}NO_3$ $[M+H]^+$ 244.0974, found: 244.0963.

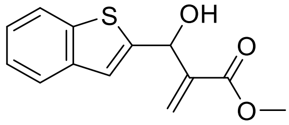

3p

Methyl 2-(benzo[b]thiophen-2-yl(hydroxy)methyl)acrylate (**3p**). Yellow solid (129 mg from **1p**, isolated yield 52%). M.p. 91~92 °C. ^{1}H NMR ($CDCl_3$, 500 MHz) δ 7.80 (d, J = 7.7 Hz, 1H), 7.71 (d, J = 7.7 Hz, 1H), 7.35-7.29 (m, 2H), 7.20 (s, 1H), 6.42 (s, 1H),

6.02 (s, 1H), 5.83 (d, *J* = 7.0 Hz, 1H), 3.77 (s, 3H), 3.53 (d, *J* = 7.2 Hz, 1H). ^{13}C NMR ($CDCl_3$, 126 MHz) δ 166.6, 146.6, 140.8, 139.8, 139.7, 126.9, 124.42, 124.39, 123.8, 122.5, 121.3, 70.5, 52.3. ESI-MS HRMS calculated for $C_{14}H_{14}NO_3$ $[M+H]^+$ 244.0974, found: 244.0963.

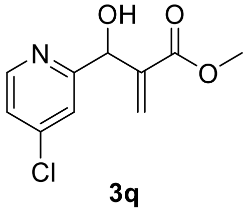

3q

Methyl 2-((4-chloropyridin-2-yl)(hydroxy)methyl)acrylate (**3q**). Yellow oil (175 mg from **1q**, isolated yield 77%). ^{1}H NMR ($CDCl_3$, 500 MHz) δ 8.39 (d, *J* = 5.4 Hz, 1H), 7.46 (s, 1H), 7.19 (dd, *J* = 5.2 Hz, 1.3 Hz, 1H), 6.36 (s, 1H), 5.95 (s, 1H), 5.55 (s, 1H), 4.66 (brs, 1H), 3.71 (s, 3H). ^{13}C NMR ($CDCl_3$, 126 MHz) δ 166.5, 161.7, 149.3, 145.1, 141.1, 127.6, 123.2, 121.8, 72.5, 52.1. ESI-MS HRMS calculated for $C_{10}H_{11}ClNO_3$ $[M+H]^+$ 228.0427, found: 228.0423.

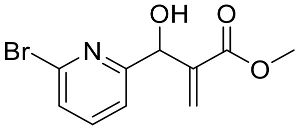

3r

Methyl 2-((6-bromopyridin-2-yl)(hydroxy)methyl)acrylate (**3r**). Yellow oil (182 mg from **1r**, isolated yield 67%). ^{1}H NMR ($CDCl_3$, 500 MHz) δ 7.52 (t, *J* = 7.8 Hz, 1H), 7.40-7.37 (m, 2H), 6.36 (s, 1H), 5.95 (s, 1H), 5.57 (d, *J* = 6.4 Hz, 1H), 4.32 (d, *J* = 6.8 Hz, 1H), 3.72 (s, 3H). ^{13}C NMR ($CDCl_3$, 126 MHz) δ 166.6, 161.5, 141.2, 141.0, 139.3, 127.6, 127.2, 120.2, 72.3, 52.1. ESI-MS HRMS calculated for $C_{10}H_{11}BrNO_3$ $[M+H]^+$ 271.9922, found: 271.9918.

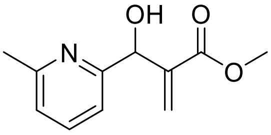

3s

Methyl 2-(hydroxy(6-methylpyridin-2-yl)methyl)acrylate (**3s**). White solid (161 mg from **1s**, isolated yield 78%). M.p. 80~82 °C. ^{1}H NMR ($CDCl_3$, 500 MHz) δ 7.52 (t, *J*

= 7.8 Hz, 1H), 7.14 (d, *J* = 7.8 Hz, 1H), 7.03 (d, *J* = 7.7 Hz, 1H), 6.31 (d, *J* = 0.4 Hz, 1H), 5.92 (s, 1H), 5.59 (s, 1H), 5.26 (brs, 1H), 3.72 (s, 3H), 2.52 (s, 3H). ^{13}C NMR ($CDCl_3$, 126 MHz) δ 166.7, 158.5, 157.2, 142.3, 137.2, 126.7, 122.2, 118.1, 71.4, 51.9, 24.3.IR (KBr): 3428, 3080, 2900, 1723, 1636, 1599, 1467, 1435, 1323, 1283, 1199, 1159, 1055, 992, 750, 711cm^{-1} ESI-MS HRMS calculated for $C_{11}H_{14}NO_3$ $[M+H]^+$ 208.0974, found: 208.0965.

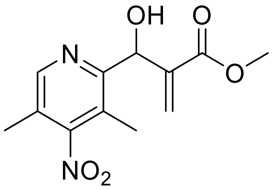

3t

Methyl 2-((3,5-dimethyl-4-nitropyridin-2-yl)(hydroxy)methyl)acrylate (**3t**). Yellow solid (170 mg from **1t**, isolated yield 64%). M.p. 83~84 ^{o}C. NMR ($CDCl_3$, 500 MHz) δ 8.43 (s, 1H), 6.34 (s, 1H), 5.73 (d, *J* = 4.8 Hz, 1H), 5.60 (s, 1H), 4.66 (d, *J* = 6.5 Hz, 1H), 3.76 (s, 3H), 2.29 (s, 3H), 2.21 (s, 3H). ^{13}C NMR ($CDCl_3$, 126 MHz) δ 166.4, 158.2, 157.9, 148.4, 141.3, 127.1, 123.0, 121.2, 69.6, 52.2, 14.1, 12.4. ESI-MS HRMS calculated for $C_{12}H_{15}N_2O_5$ $[M+H]^+$ 267.0981, found: 267.0974.

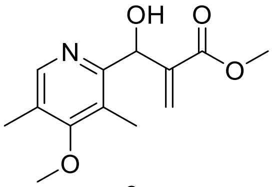

3u

Methyl 2-(hydroxy(4-methoxy-3,5-dimethylpyridin-2-yl)methyl)acrylate (**3u**). Yellow solid (156 mg from **1u**, isolated yield 62%). M.p. 76~78 ^{o}C. ^{1}H NMR ($CDCl_3$, 500 MHz) δ 8.22 (s, 1H), 6.24 (s, 1H), 5.67 (s, 1H), 5.39 (s, 1H), 3.77 (s, 3H), 3.75 (s, 3H), 2.26 (s, 3H), 2.13 (s, 3H). ^{13}C NMR ($CDCl_3$, 126 MHz) δ 166.9, 164.5, 156.9, 148.1, 142.3, 126.5, 125.9, 123.9, 68.7, 60.1, 52.1, 13.4, 10.6. ESI-MS HRMS calculated for $C_{13}H_{18}NO_4$ $[M+H]^+$ 252.1236, found: 252.1230.

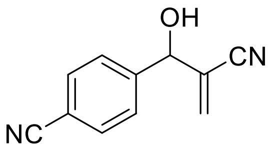

3v

4-(2-cyano-1-hydroxyallyl)benzonitrile (**3v**). Yellow oil (110 mg from **1v**, isolated yield 60%). ^{1}H NMR ($CDCl_3$, 500 MHz) δ 7.65 (d, *J* = 8.1 Hz, 2H), 7.52 (d, *J* = 8.0 Hz, 2H), 6.13 (s, 1H), 6.05 (s, 1H), 5.35 (s, 1H), 3.80 (brs, 1H). ^{13}C NMR ($CDCl_3$, 126 MHz) δ 144.5, 132.8, 130.9, 127.3, 125.7, 118.5, 116.5, 112.7, 73.5. ESI-MS HRMS calculated for $C_{11}H_9N_2O$ $[M+H]^+$ 185.0715, found: 185.0720.

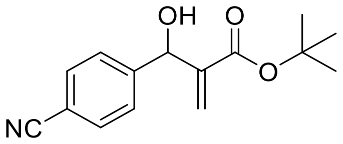

3w

Tert-butyl 2-((4-cyanophenyl)(hydroxy)methyl)acrylate (**3w**). Yellow solid (181 mg from **1w**, isolated yield 70%). M.p. 54~56 °C. ^{1}H NMR ($CDCl_3$, 500 MHz) δ 7.64 (d, *J* = 8.2 Hz, 2H), 7.50 (d, *J* = 8.2 Hz, 2H), 6.28 (s, 1H), 5.73 (s, 1H), 5.51 (d, *J* = 6.1 Hz, 1H), 3.42 (d, *J* = 6.4 Hz, 1H), 1.41 (s, 9H). ^{13}C NMR ($CDCl_3$, 126 MHz) δ 165.4, 147.2, 142.6, 132.3, 127.3, 126.4, 118.9, 111.6, 82.4, 73.3, 28.1. ESI-MS HRMS calculated for $C_{15}H_{18}NO_3$ $[M+H]^+$ 260.1287, found: 260.1280.

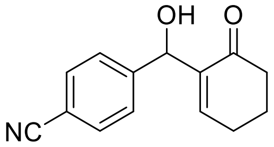

3x

4-(hydroxy(6-oxocyclohex-1-en-1-yl)methyl)benzonitrile (**3x**). Yellow oil (159 mg from **1x**, isolated yield 70%). ^{1}H NMR ($CDCl_3$, 500 MHz) δ 7.59 (d, *J* = 8.2 Hz, 2H), 7.46 (d, *J* = 8.2 Hz, 2H), 6.82 (t, *J* = 4.1 Hz, 1H), 5.54 (d, *J* = 4.9 Hz, 1H), 3.68 (d, *J* = 5.7 Hz, 1H), 2.43-2.39 (m, 4H), 2.01-1.95 (m, 2H). ^{13}C NMR ($CDCl_3$, 126 MHz) δ 200.0, 148.0, 147.6, 140.4, 132.1, 127.1, 118.9, 111.1, 71.7, 38.4, 25.8, 22.4. ESI-MS HRMS calculated for $C_{14}H_{14}NO_2$ $[M+H]^+$ 228.1025, found: 228.1024.

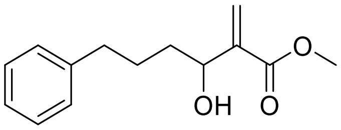

3y

Methyl 3-hydroxy-2-methylene-6-phenylhexanoate (**3y**). Colorless oil (145 mg from **1y**, isolated yield 62%). ^{1}H NMR ($CDCl_3$, 500 MHz) δ 7.28 (t, *J* = 7.5 Hz, 2H), 7.19-7.18 (m, 3H), 6.23 (s, 1H), 5.79 (s, 1H), 4.43 (s, 1H), 3.77(s, 3H), 2.67-2.65 (m, 3H), 1.82-1.67 (m, 4H). ^{13}C NMR ($CDCl_3$, 126 MHz) δ 167.1, 142.5, 142.3, 128.5, 128.4, 125.8, 125.1, 71.5, 51.9, 35.8, 35.7, 27.6. IR (KBr): 3437, 3001, 2949, 1717, 1629, 1603, 1453, 1439, 1398, 1287, 1195, 1157, 1087, 991, 750, 700 cm^{-1} ESI-MS HRMS calculated for $C_{14}H_{19}O_3$ $[M+H]^+$ 235.1335, found: 235.1321.

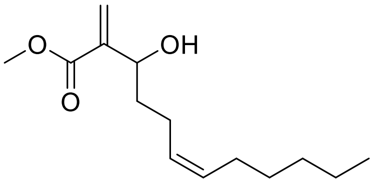

3z

Methyl (S,Z)-3-hydroxy-2-methylenedodec-6-enoate (**3z**). Colorless oil (154 mg from **1z**, isolated yield 64%). ^{1}H NMR ($CDCl_3$, 500 MHz) δ 6.23 (s, 1H), 5.81 (s, 1H), 5.46-5.34 (m, 2H), 4.41 (t, *J* = 6.5 Hz, 1H), 3.78 (s, 3H), 2.58 (brs, 1H), 2.22-2.10 (m, 2H), 2.05-2.01 (m, 2H), 1.73-1.67 (m, 2H), 1.36-1.26 (m, 6H), 0.88 (t, *J* = 6.7 Hz, 3H). ^{13}C NMR ($CDCl_3$, 126 MHz) δ 167.1, 142.5, 131.2, 128.7, 125.2, 71.5, 52.0, 36.2, 31.6, 29.5, 27.3, 23.8, 22.7, 14.2. ESI-MS HRMS calculated for $C_{14}H_{25}O_3$ $[M+H]^+$ 241.1804, found: 241.1896.

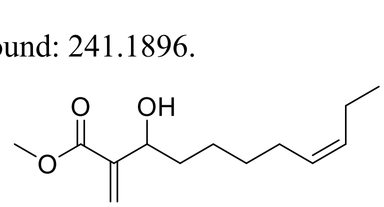

3aa

Methyl (S,Z)-3-hydroxy-2-methyleneundec-8-enoate (**3aa**). Colorless oil (152 mg from **1aa**, isolated yield 67%). ^{1}H NMR ($CDCl_3$, 500 MHz) δ 6.21 (d, *J* = 0.8 Hz, 1H), 5.78 (t, *J* = 1.1 Hz, 1H), 5.37-5.27 (m, 2H), 4.38 (dd, *J* = 7.1 Hz, 5.9 Hz, 1H), 3.77 (s, 3H), 2.62 (brs, 1H), 2.04-1.96 (m, 4H), 1.69-1.58 (m, 2H), 1.48-1.32 (m, 4H), 0.94 (t, *J* =

7.6 Hz, 3H). ^{13}C NMR ($CDCl_3$, 126 MHz) δ 167.1, 142.7, 131.9, 129.0, 125.0, 71.8, 51.9, 36.3, 29.6, 27.1, 25.6, 20.6, 14.4. ESI-MS HRMS calculated for $C_{13}H_{23}O_3$ $[M+H]^+$ 227.1647, found: 227.1638.

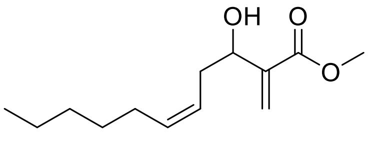

3ab

Methyl (Z)-3-hydroxy-2-methyleneundec-5-enoate (**3ab**). Colorless oil (147 mg from **1ab**, isolated yield 65%). ^{1}H NMR ($CDCl_3$, 500 MHz) δ 6.19 (d, J = 0.7 Hz, 1H), 5.78 (s, 1H), 5.36-5.26 (m, 2H), 4.37 (d, J = 5.0 Hz, 1H), 3.75 (s, 3H), 2.70 (d, J = 5.1 Hz, 1H), 2.03-1.97 (m, 4H), 1.68-1.56 (m, 2H), 1.48-1.29 (m, 4H), 0.92 (t, J = 7.6 Hz, 3H). ^{13}C NMR ($CDCl_3$, 126 MHz) δ 167.1, 142.7, 131.8, 129.0, 125.0, 71.6, 51.9, 36.2, 29.6, 27.0, 25.5, 20.5, 14.4. ESI-MS HRMS calculated for $C_{13}H_{23}O_3$ $[M+H]^+$ 227.1647, found: 227.1637.

5. Reference

[1] F. Schaufelberger and O. Ramstrom, *J. Am. Chem. Soc.,* 2016, **138**, 7836.

[2] A. Latorre, J. A. Saez, S. Rodríguez and F. V. Gonzalez, *Tetrahedron.* 2014, **70**, 97.

[3] P. V. Ramachandran and D. R. Nicponski, *Chem. Commun.,* 2014, **50**, 15216.

[4] X. Han, Y. Wang, F. Zhong and Y. Lu, *Org. Biomol. Chem.,* 2011, **9**, 6734.

[5] B. Zhu, L. Yan, Y. Pan, R. Lee, H. Liu, Z. Han, K.-W. Huang, C.-H. Tan and Z. Jiang, *J. Org. Chem.,* 2011, **76**, 6894.

[6] S.-R. Sheng, F.-F. Huang, S.-Y. Lin, X.-L. Liu and X. Huang, *Synthesis* 2007, **9**, 1373.

[7] C. Rasson, A. Stouse, A. Boreux, V. Cirriez and O. Riant, *Chem. Eur. J.,* 2018, **24**, 9234.

[8] G. W. Amarante, and F. Coelho, *Tetrahedron.* 2010, **66**, 6749.

6. NMR Spectra

3a, ^{1}H NMR, 500 MHz, $CDCl_3$

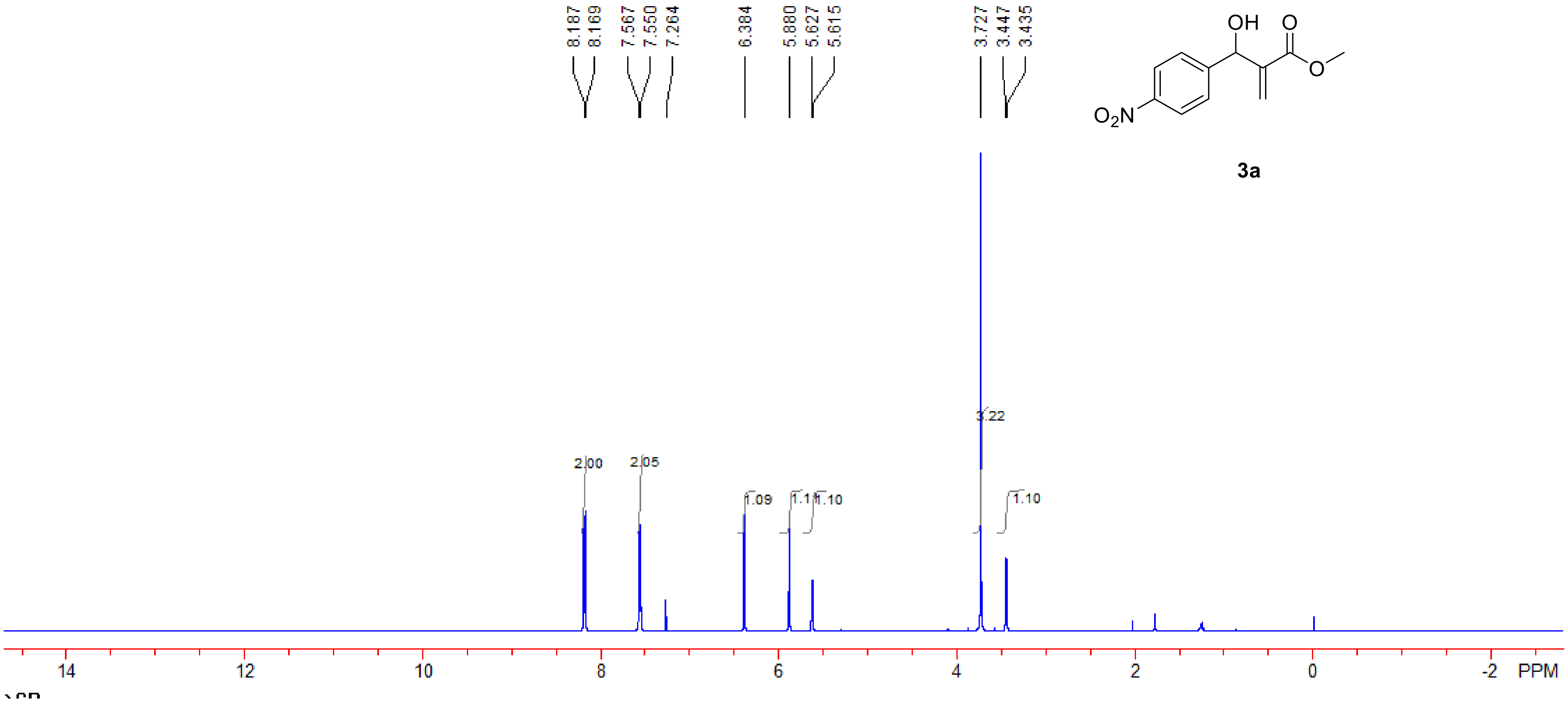

3b, ^{1}H NMR, 500 MHz, $CDCl_3$

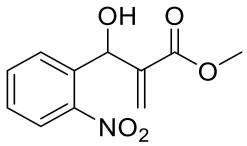

3b

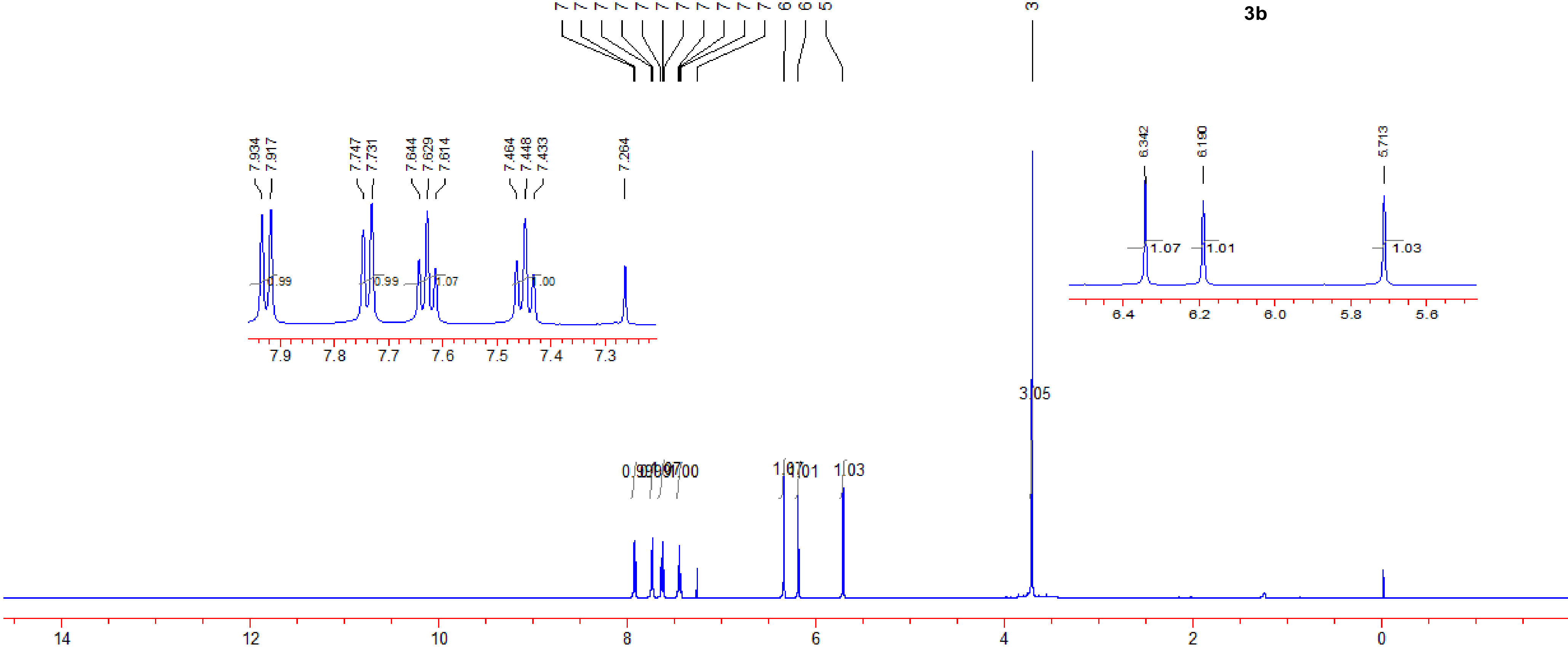

3c, ^{1}H NMR, 500 MHz, $CDCl_3$

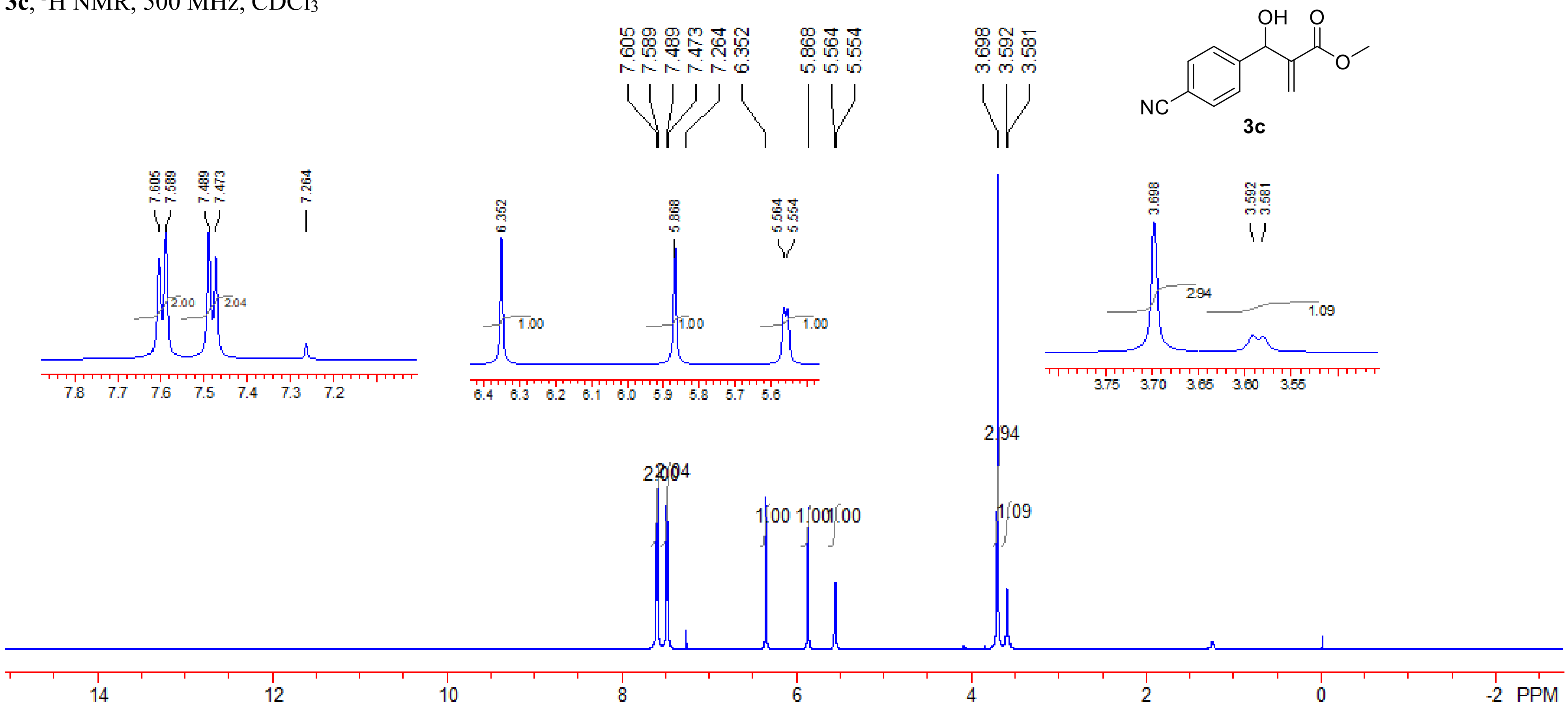

3d, ^{1}H NMR, 500 MHz, $CDCl_3$

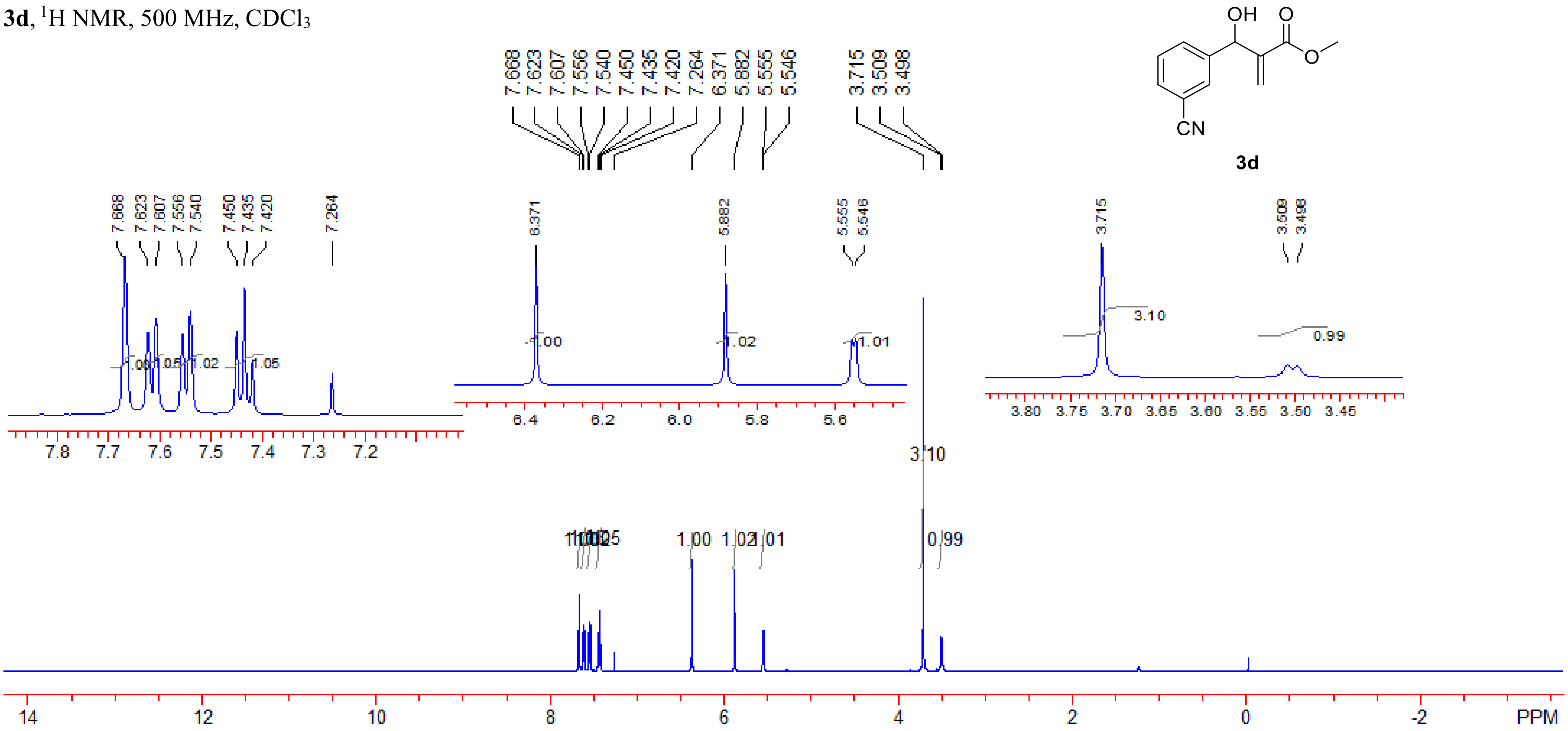

3e, ^{1}H NMR, 500 MHz, $CDCl_3$

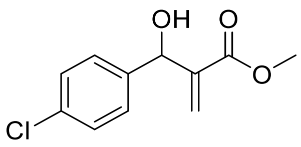

3e

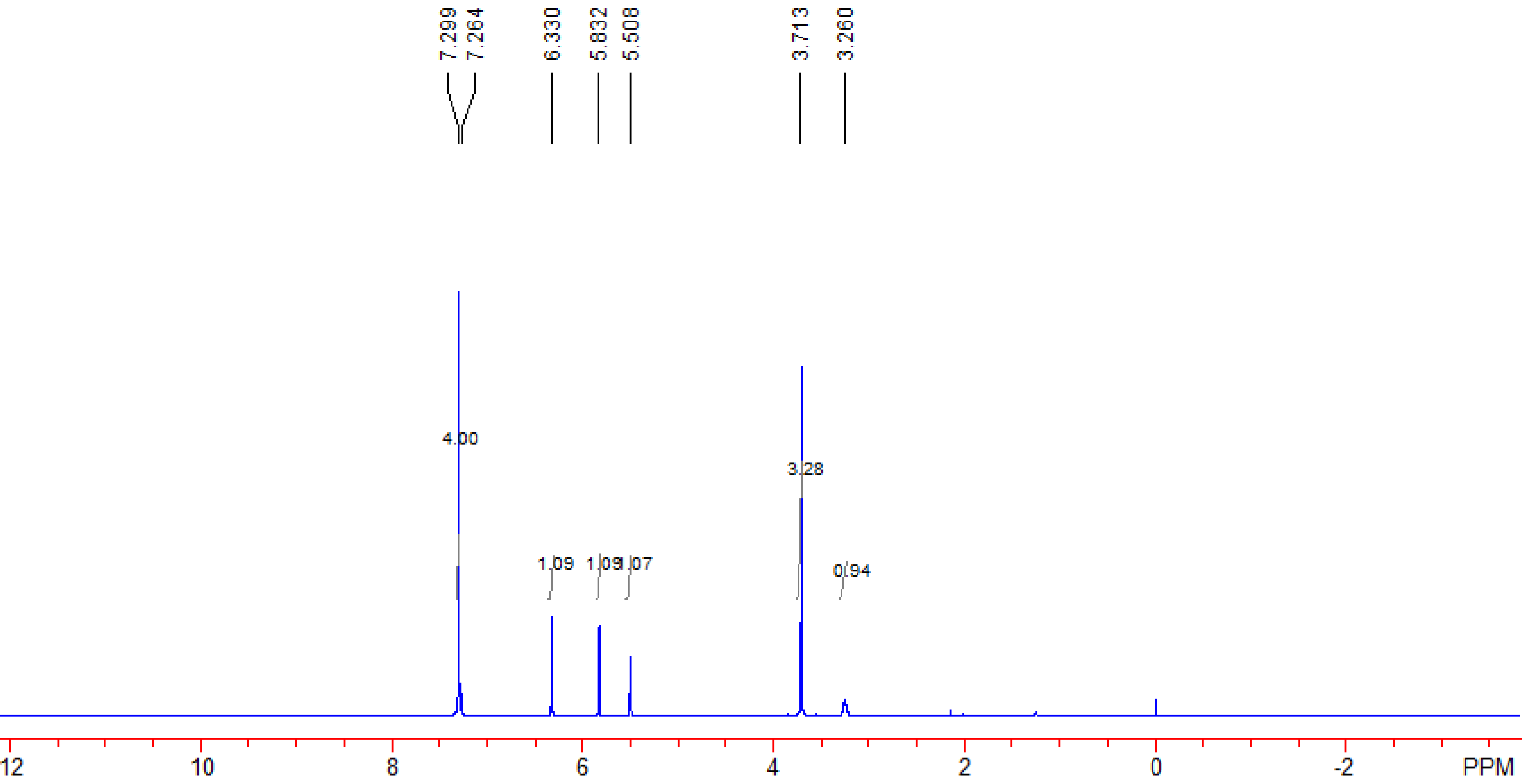

3f, ^{1}H NMR, 500 MHz, $CDCl_3$

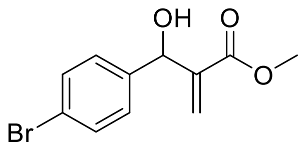

3f

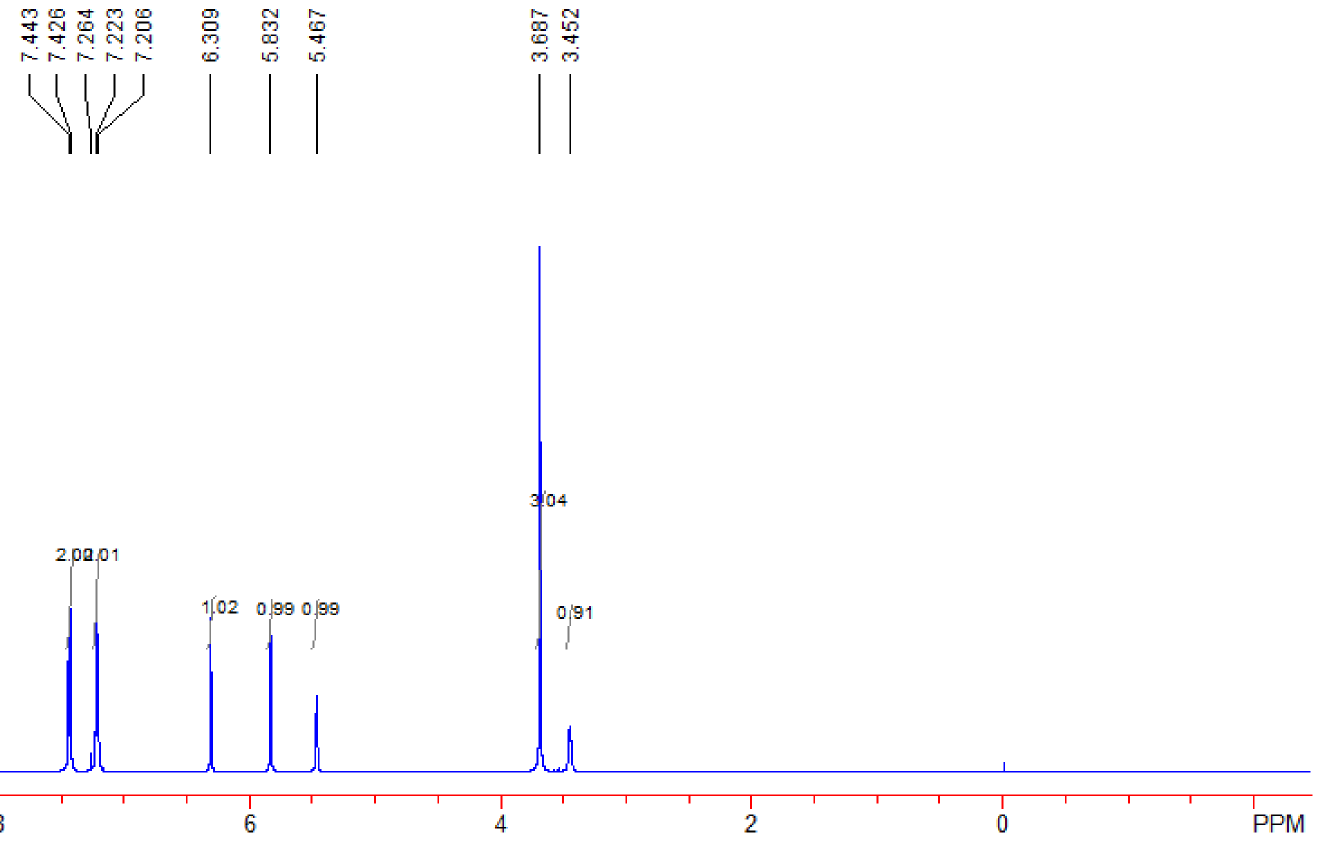

3g, ^{1}H NMR, 500 MHz, CDCl3

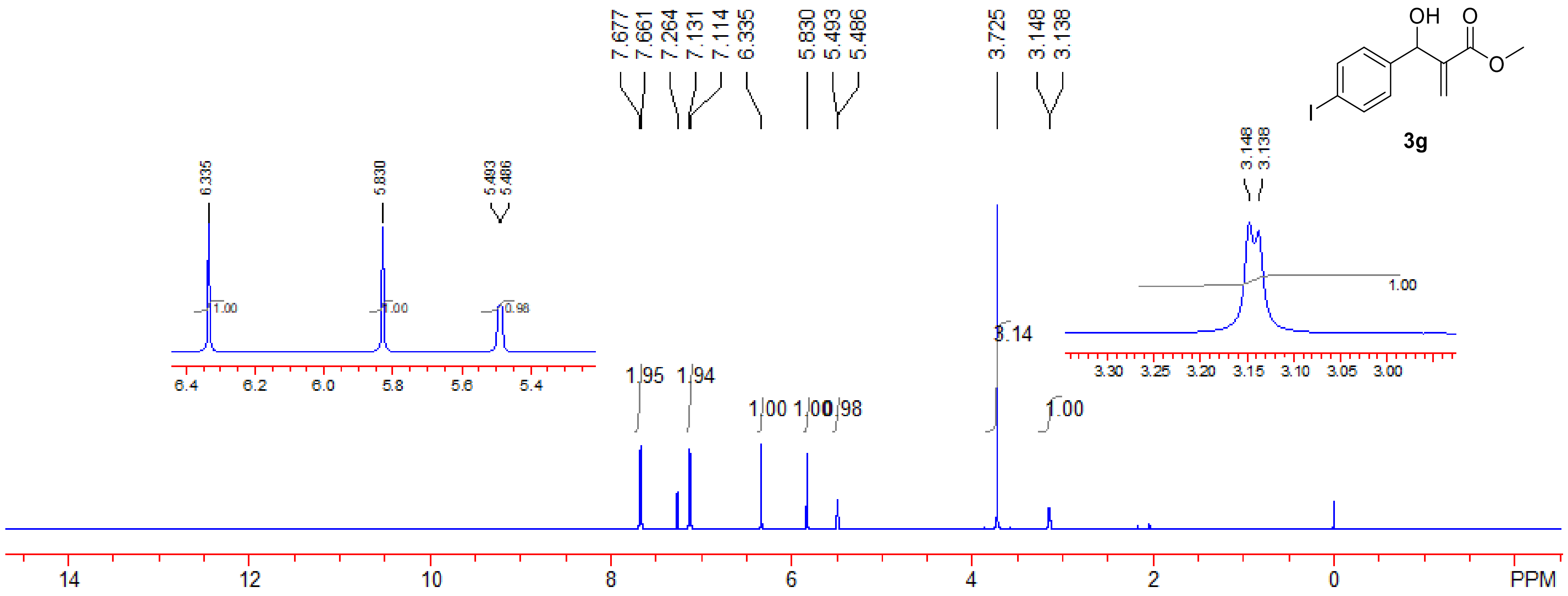

3g, ^{13}C NMR, 126 MHz, $CDCl_3$

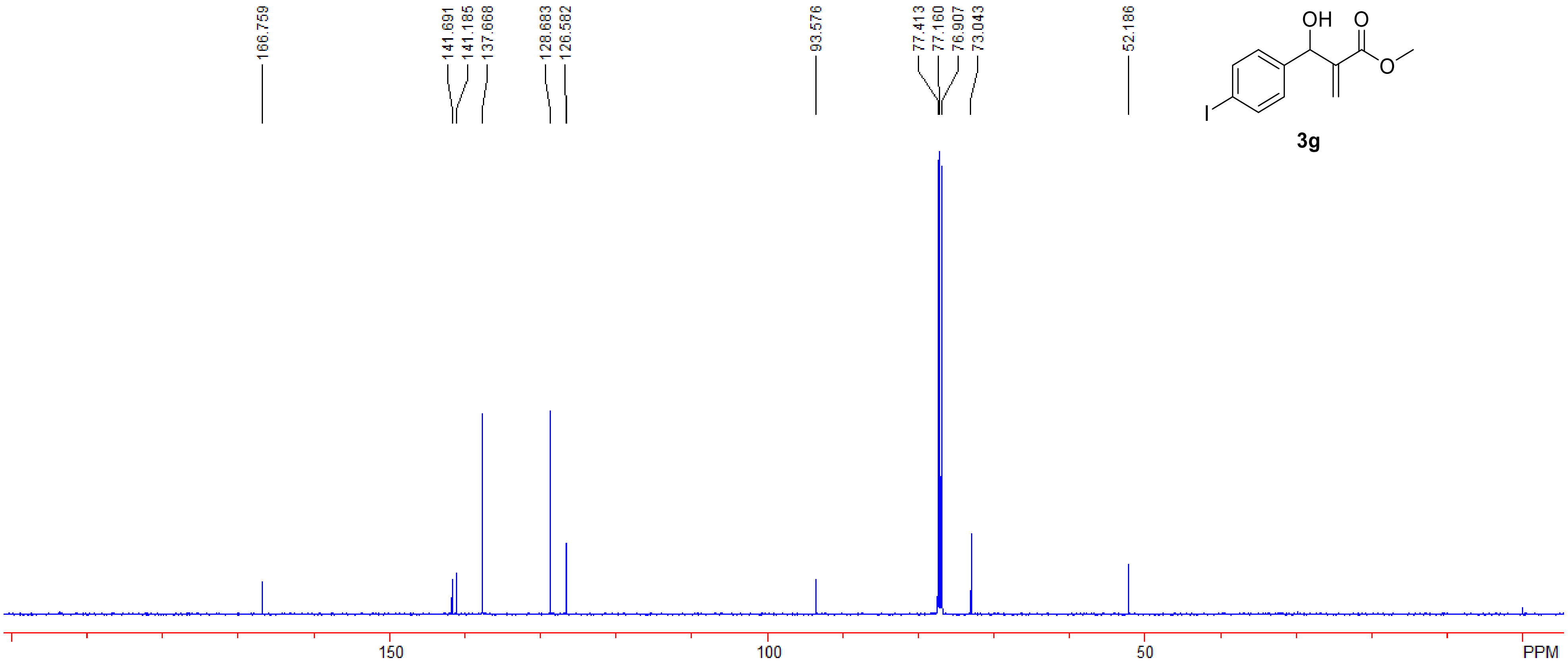

3h, ^1H NMR, 500 MHz, $CDCl_3$

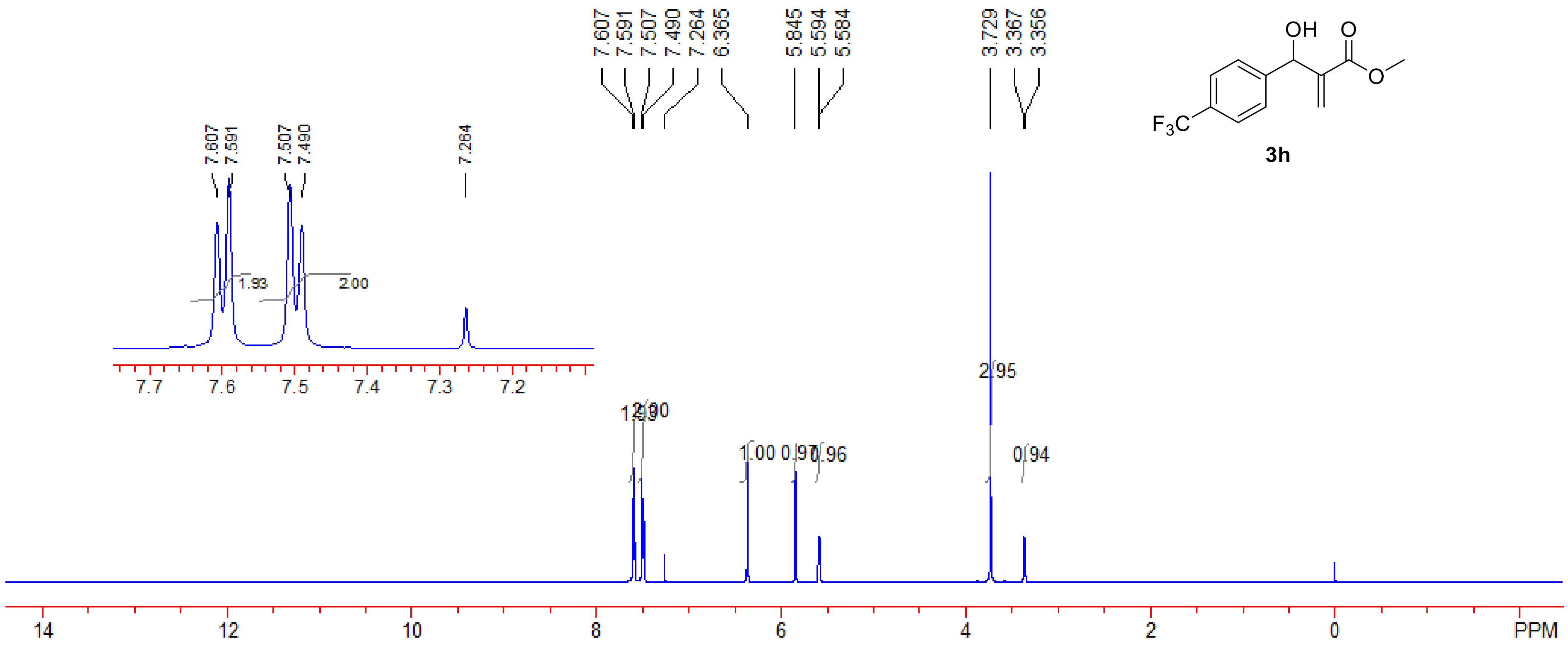

3i, ^{1}H NMR, 500 MHz, $CDCl_3$

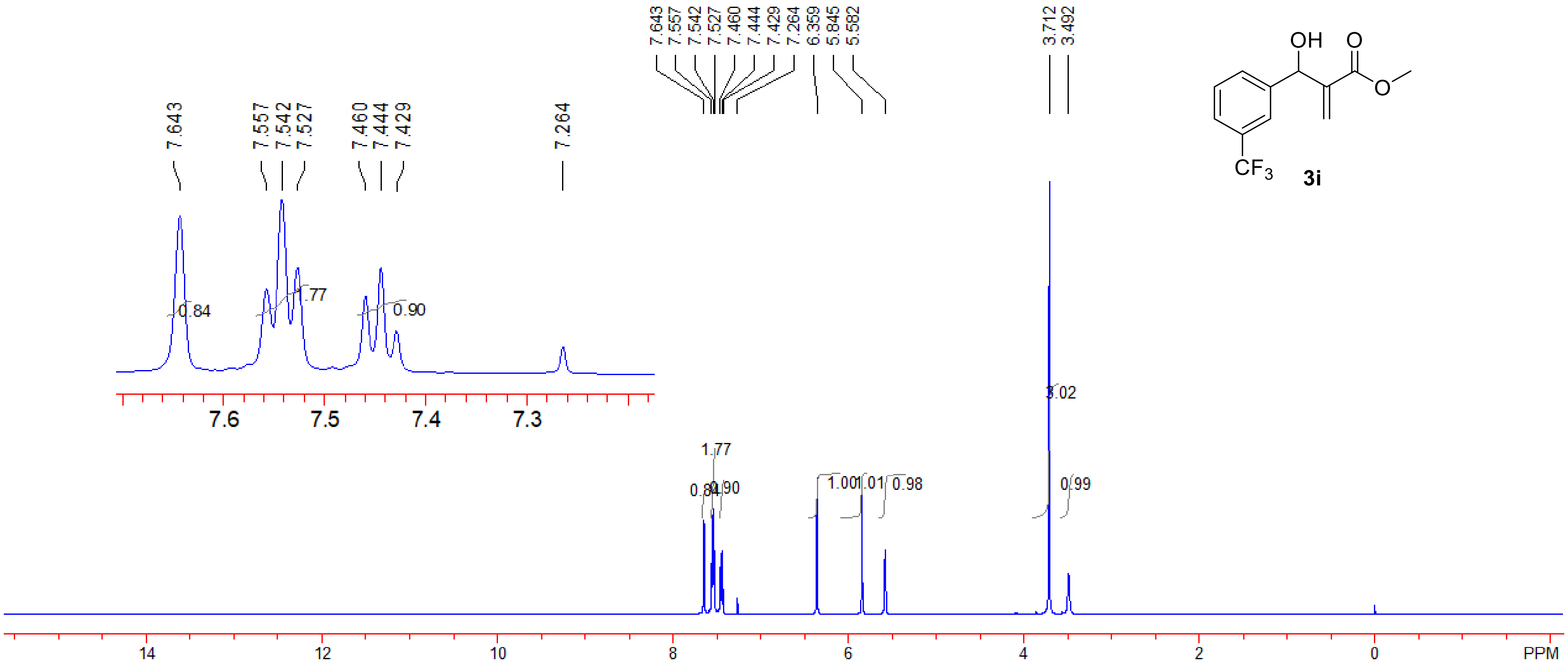

3j, ^{1}H NMR, 500 MHz, $CDCl_3$

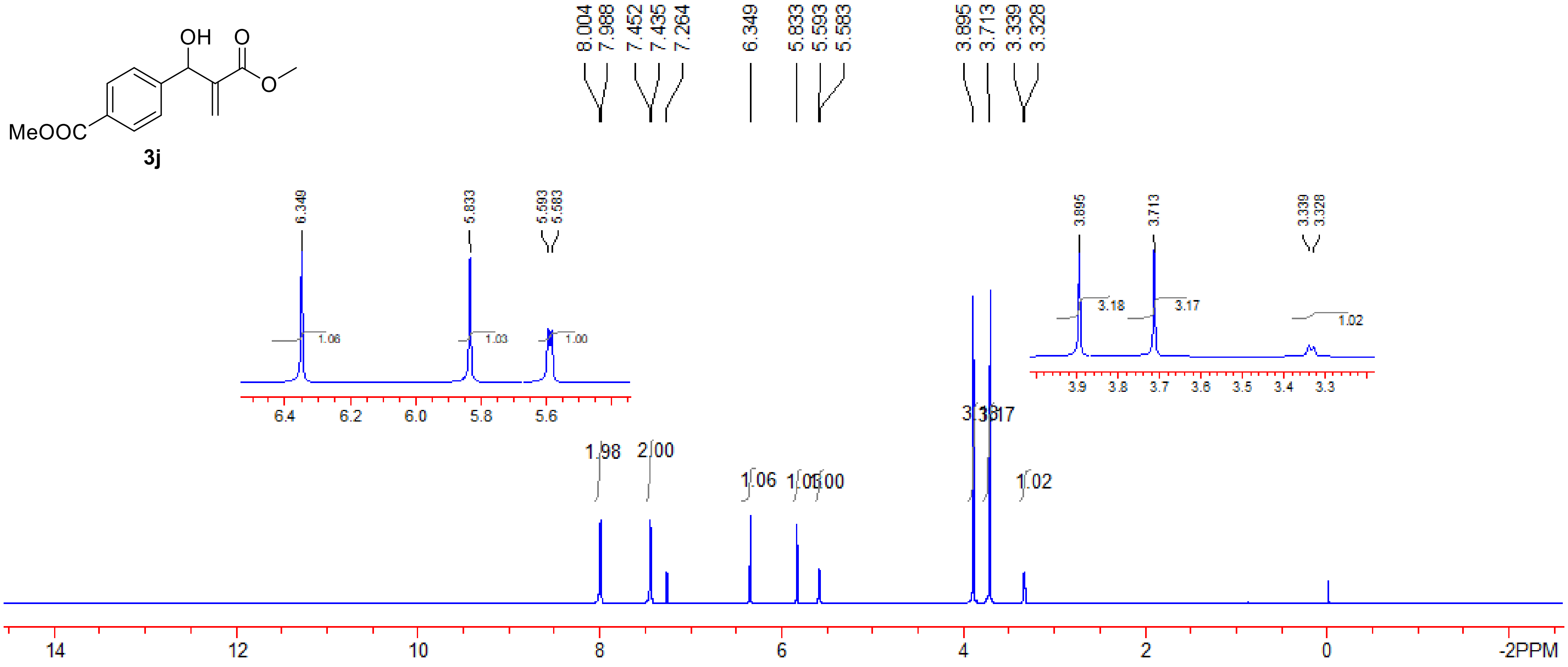

3k, ^{1}H NMR, 500 MHz, CDCl3

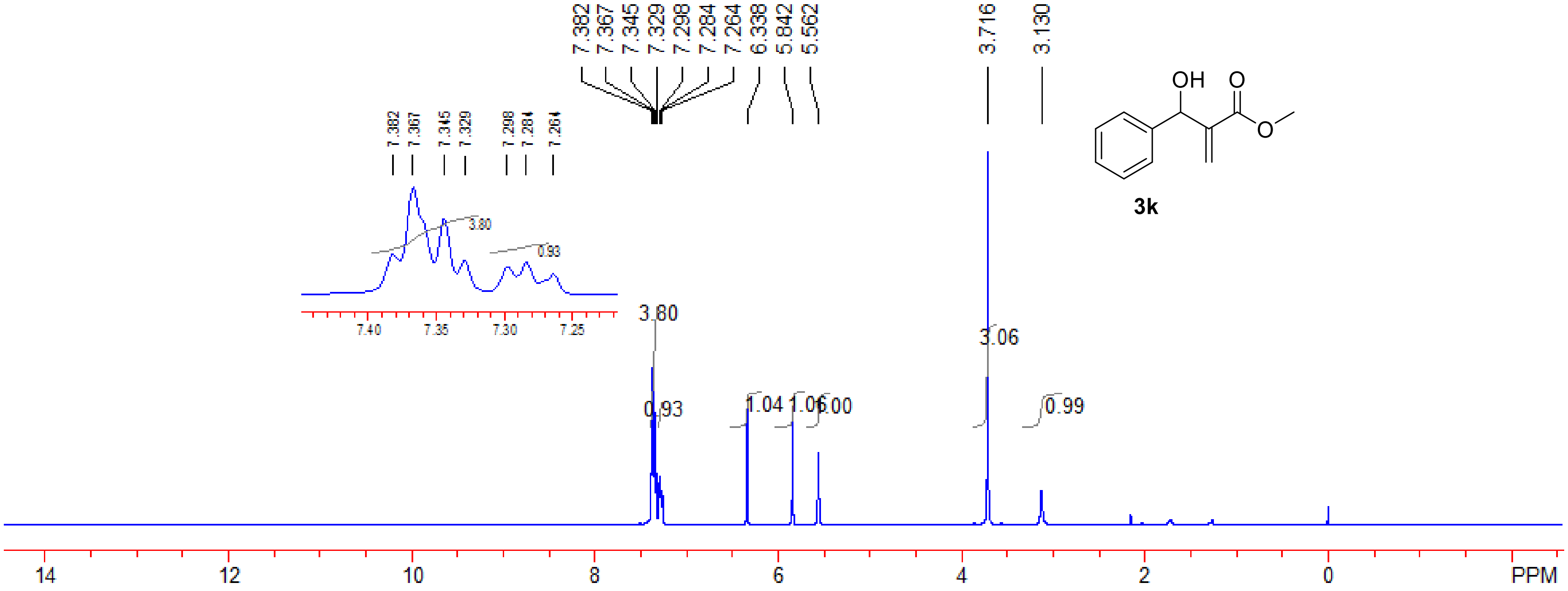

3l, ^{1}H NMR, 500 MHz, $CDCl_3$

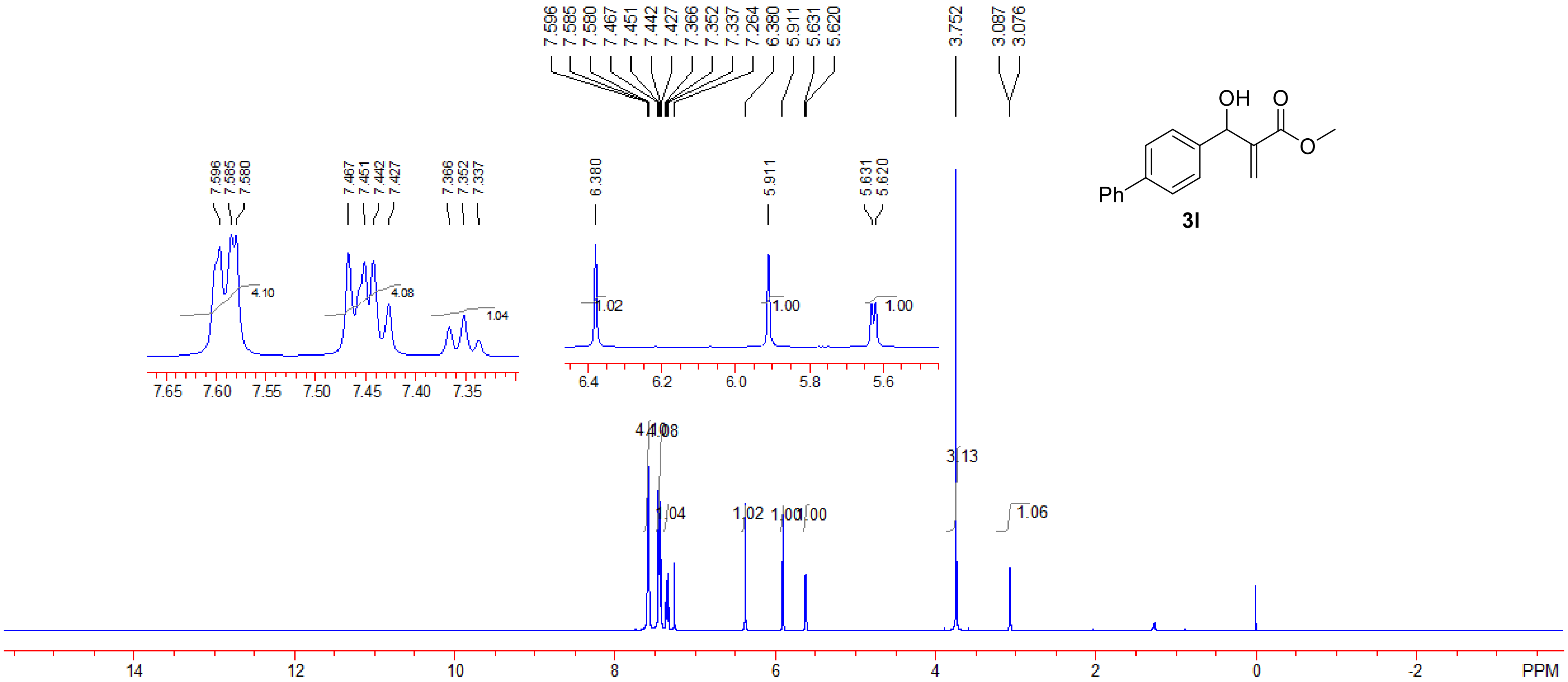

3m, ^{1}H NMR, 500 MHz, $CDCl_3$

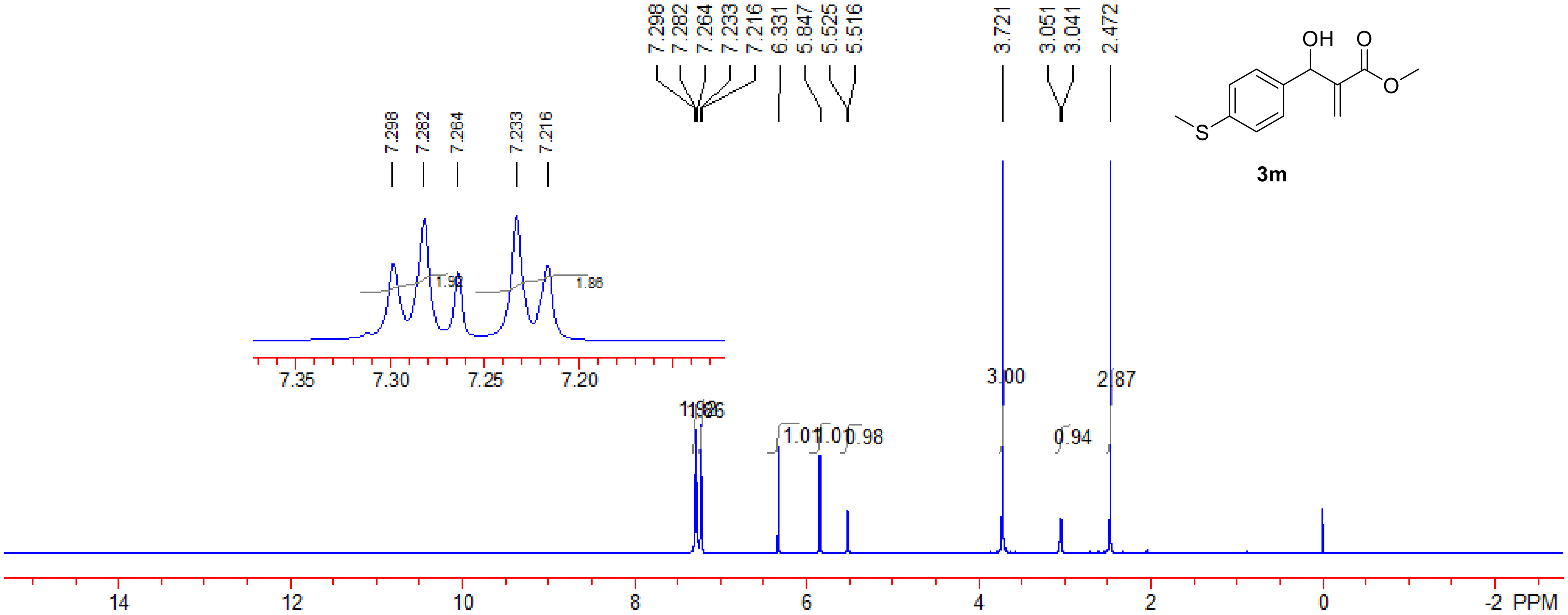

3n, ^{1}H NMR, 500 MHz, $CDCl_3$

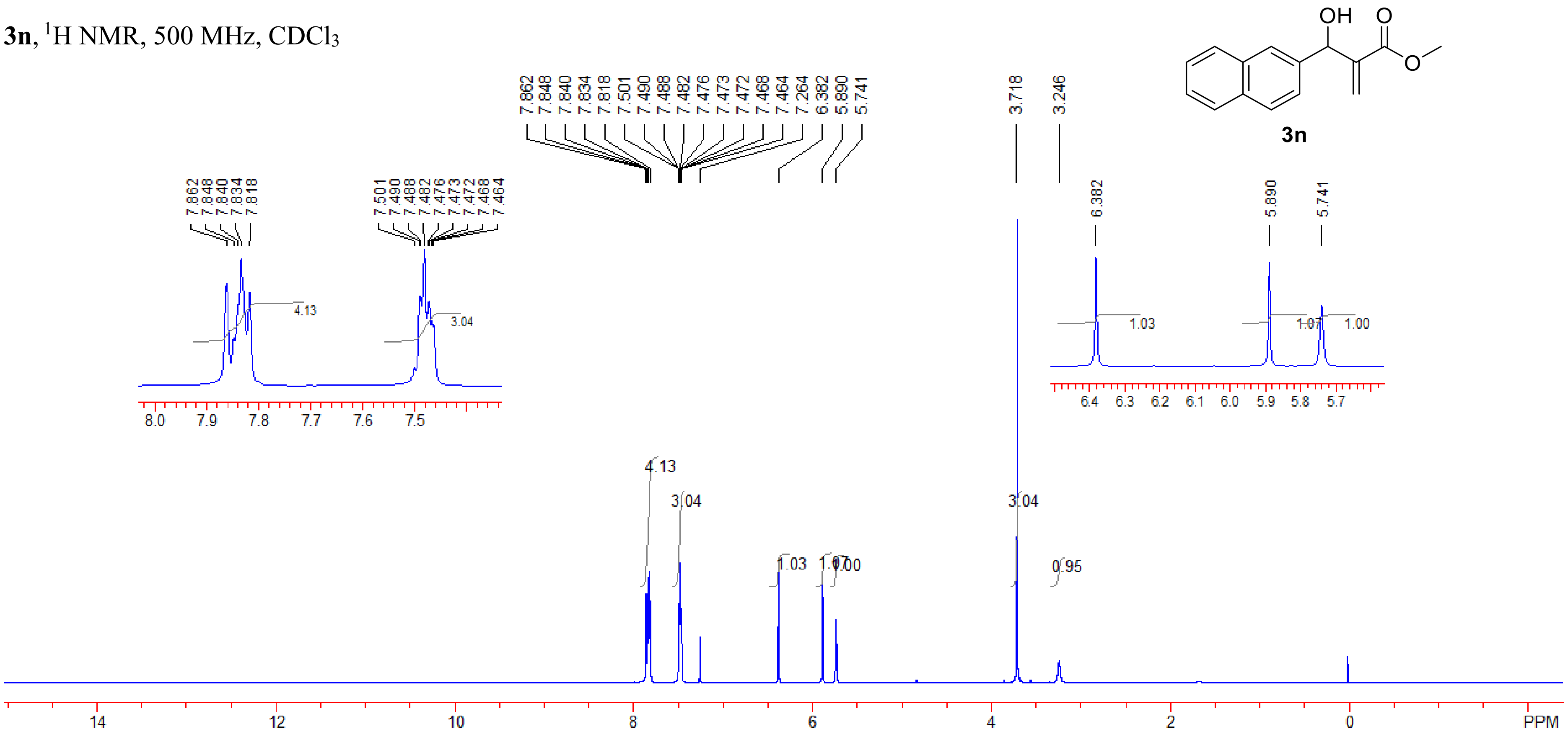

3o, ^{1}H NMR, 500 MHz, $CDCl_3$

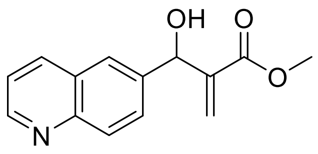

3o

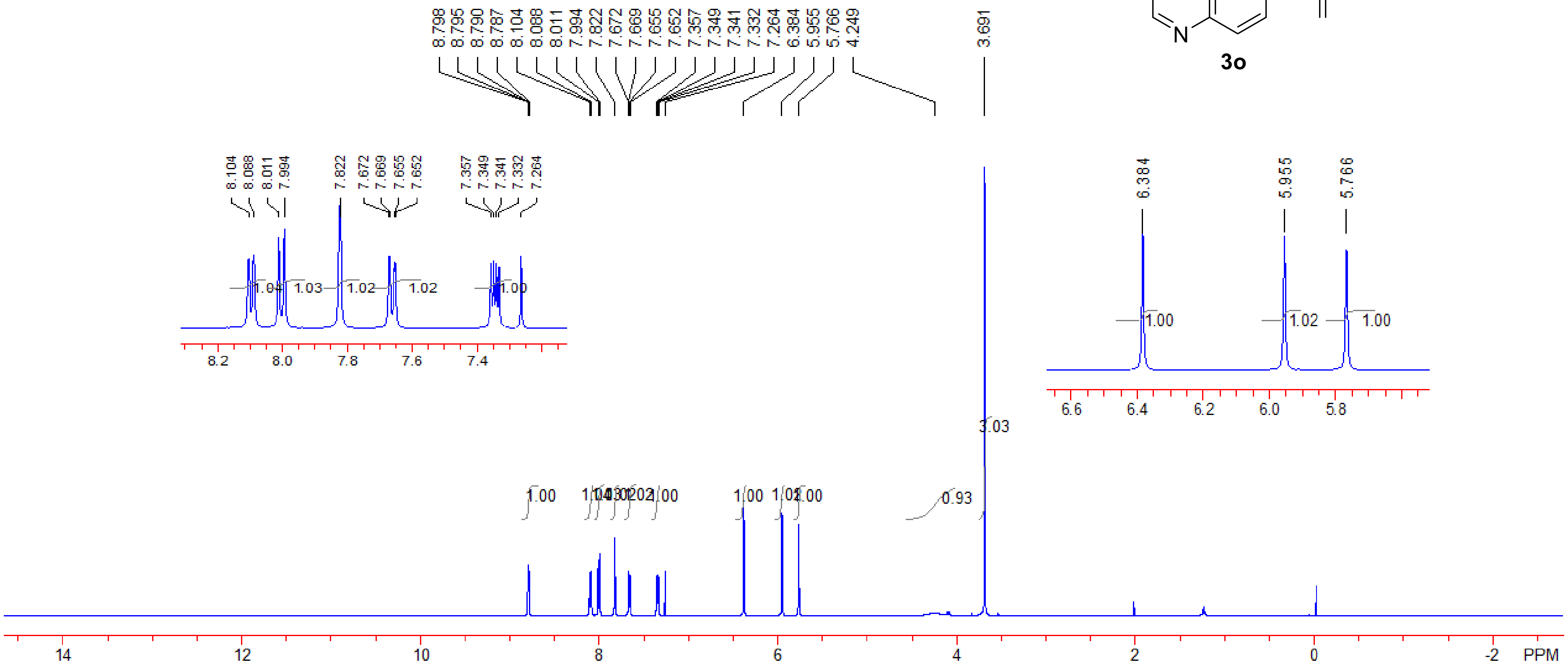

3o, ^{13}C NMR, 126 MHz, $CDCl_3$

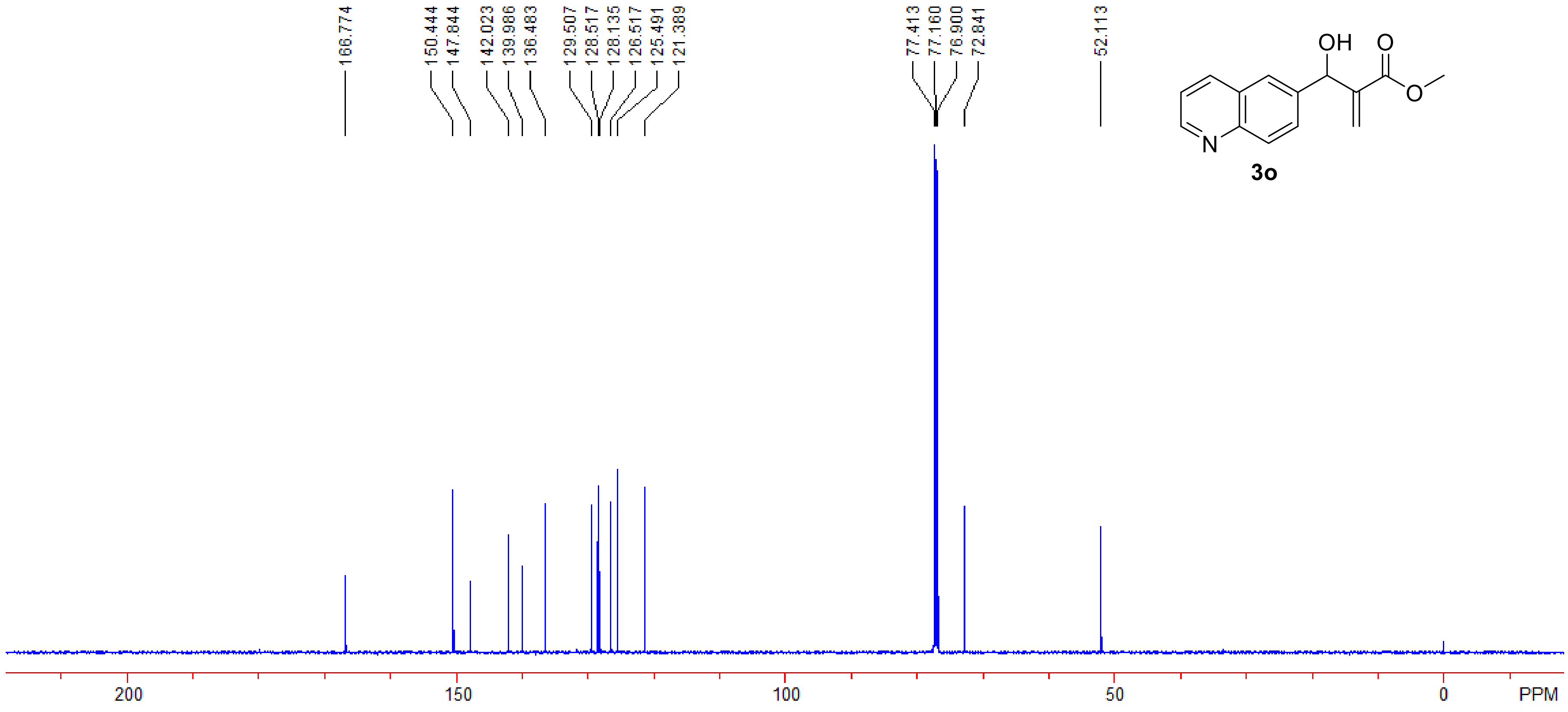

3p, ^{1}H NMR, 500 MHz, CDCl$_3$

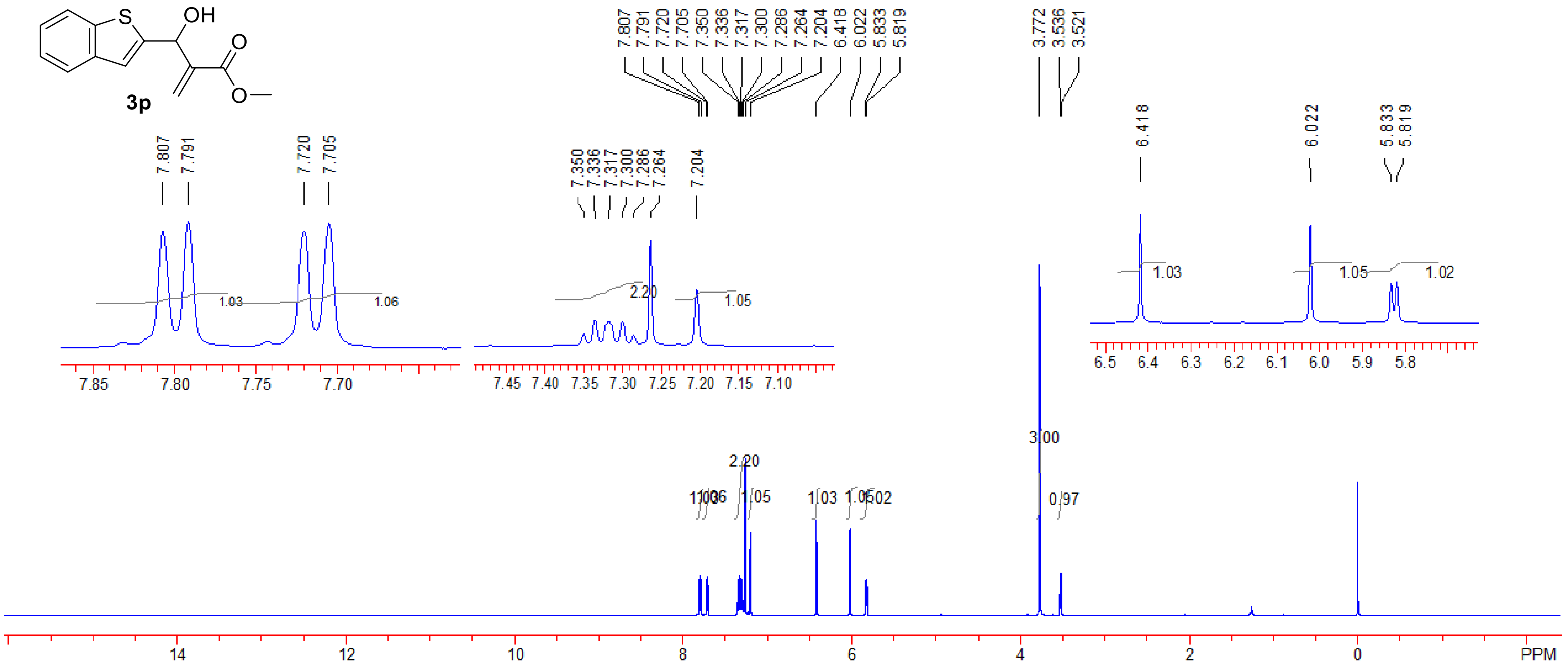

3p, ^{13}C NMR, 126 MHz, $CDCl_3$

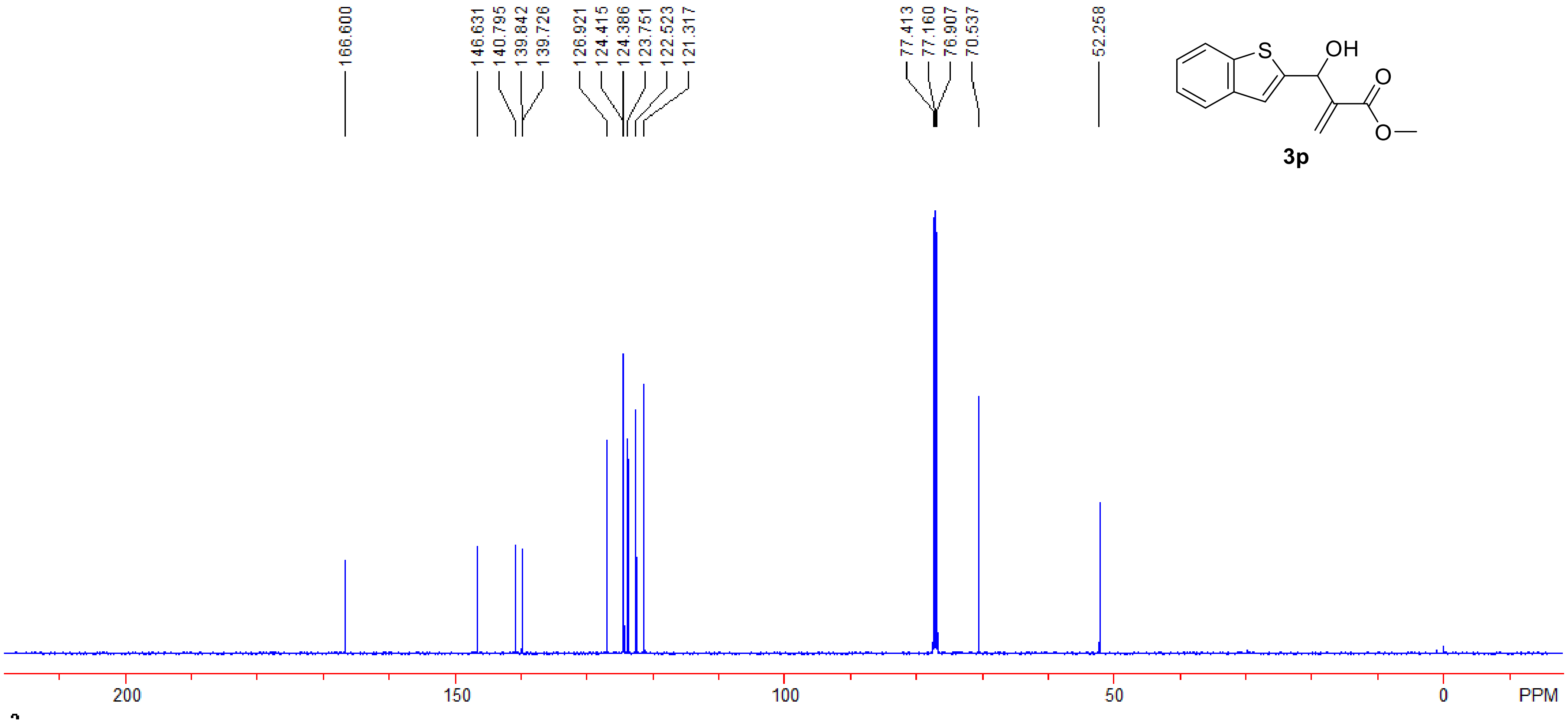

3p

3q, ^{1}H NMR, 500 MHz, $CDCl_3$

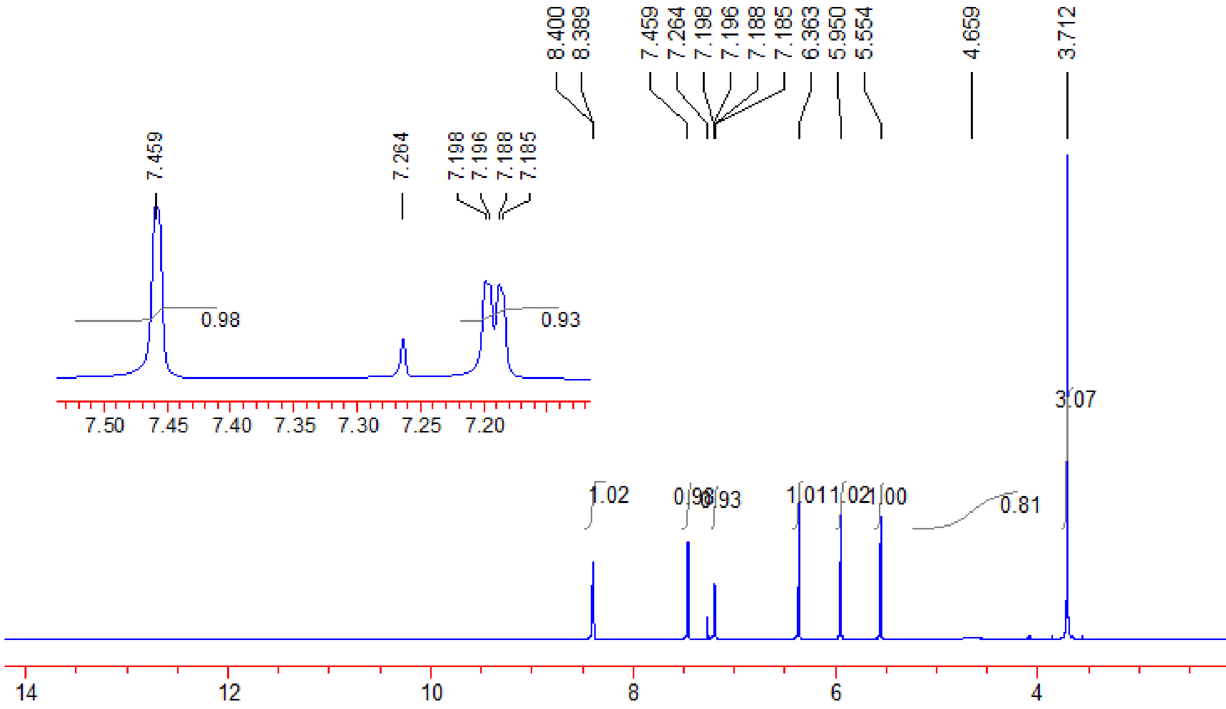

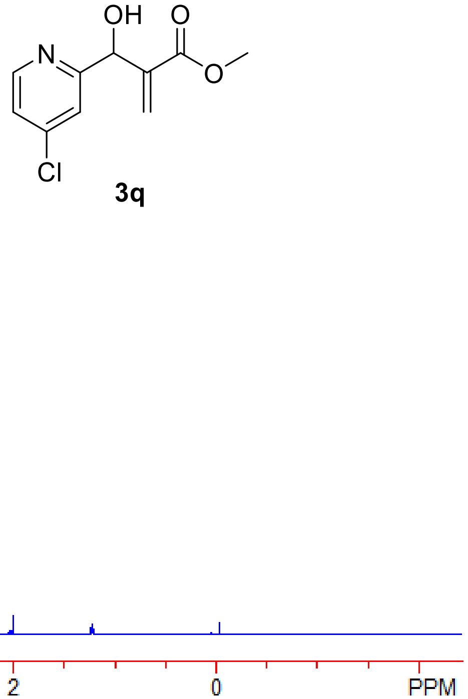

3q

3q, ^{13}C NMR, 126 MHz, $CDCl_3$

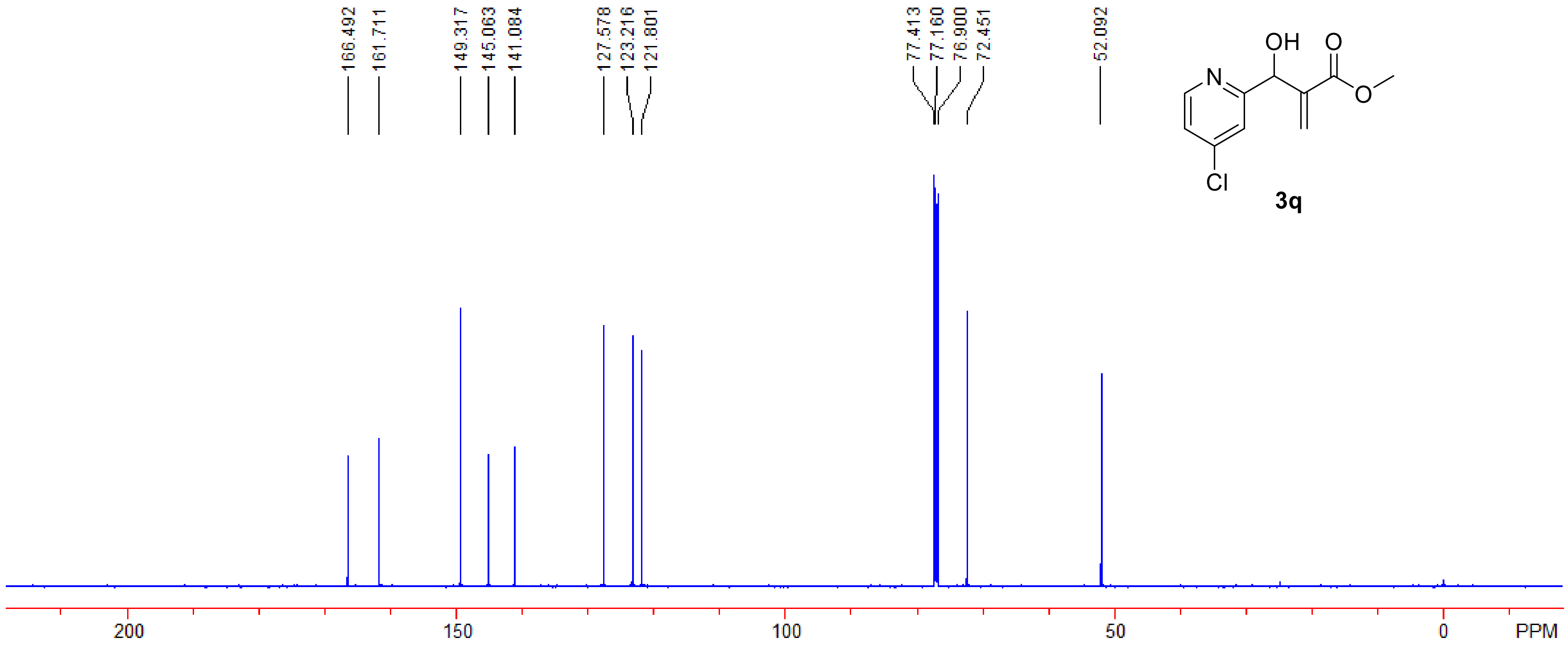

3r, ^{1}H NMR, 500 MHz, $CDCl_3$

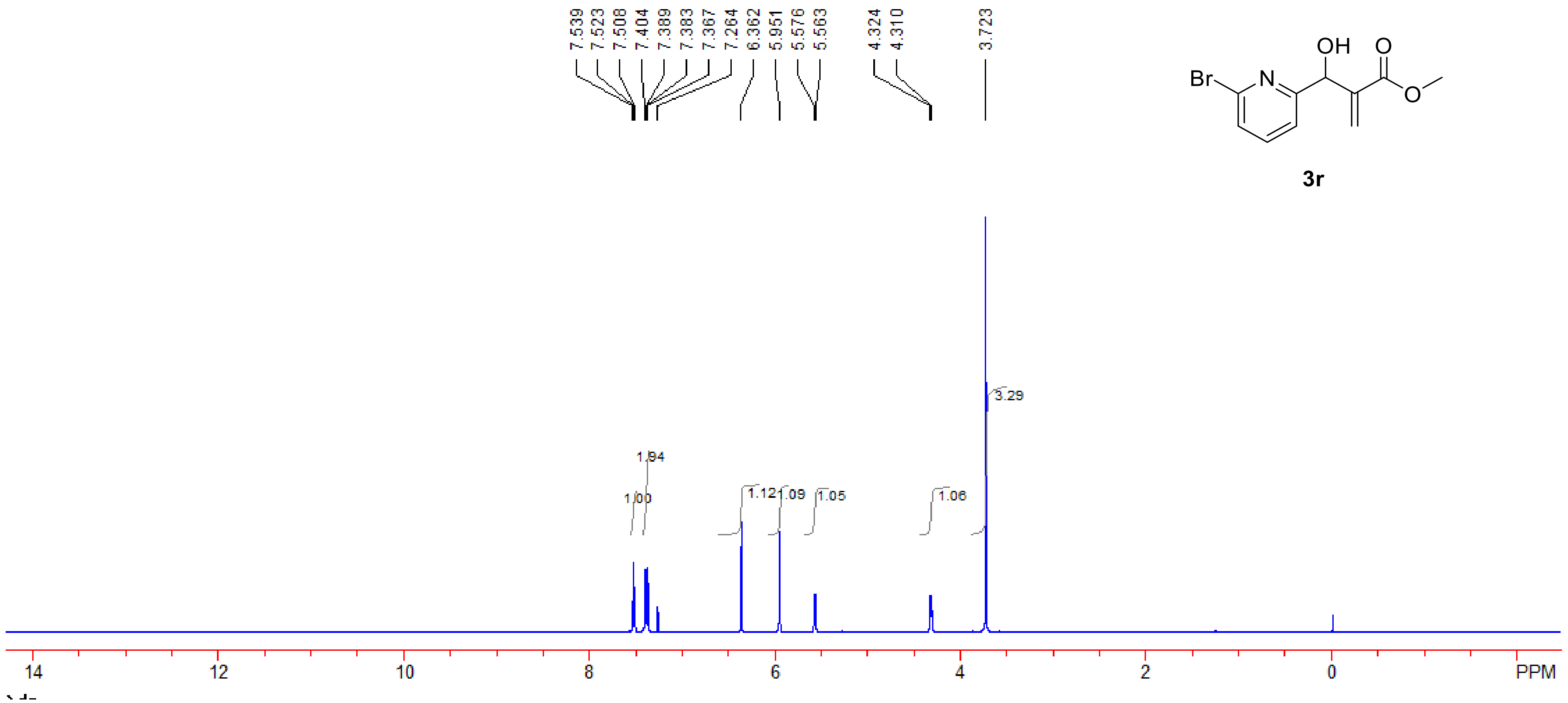

3r, ^{13}C NMR, 126 MHz, $CDCl_3$

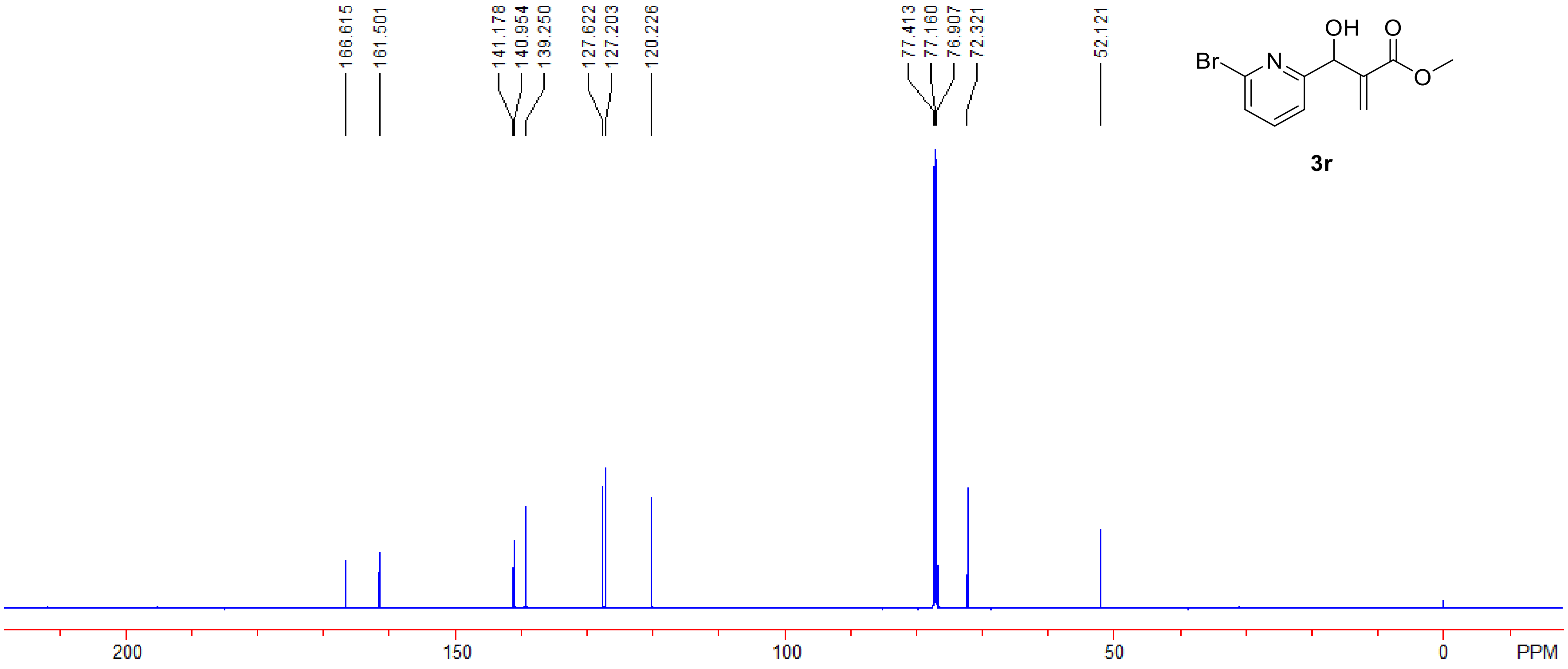

3s, ^{1}H NMR, 500 MHz, $CDCl_3$

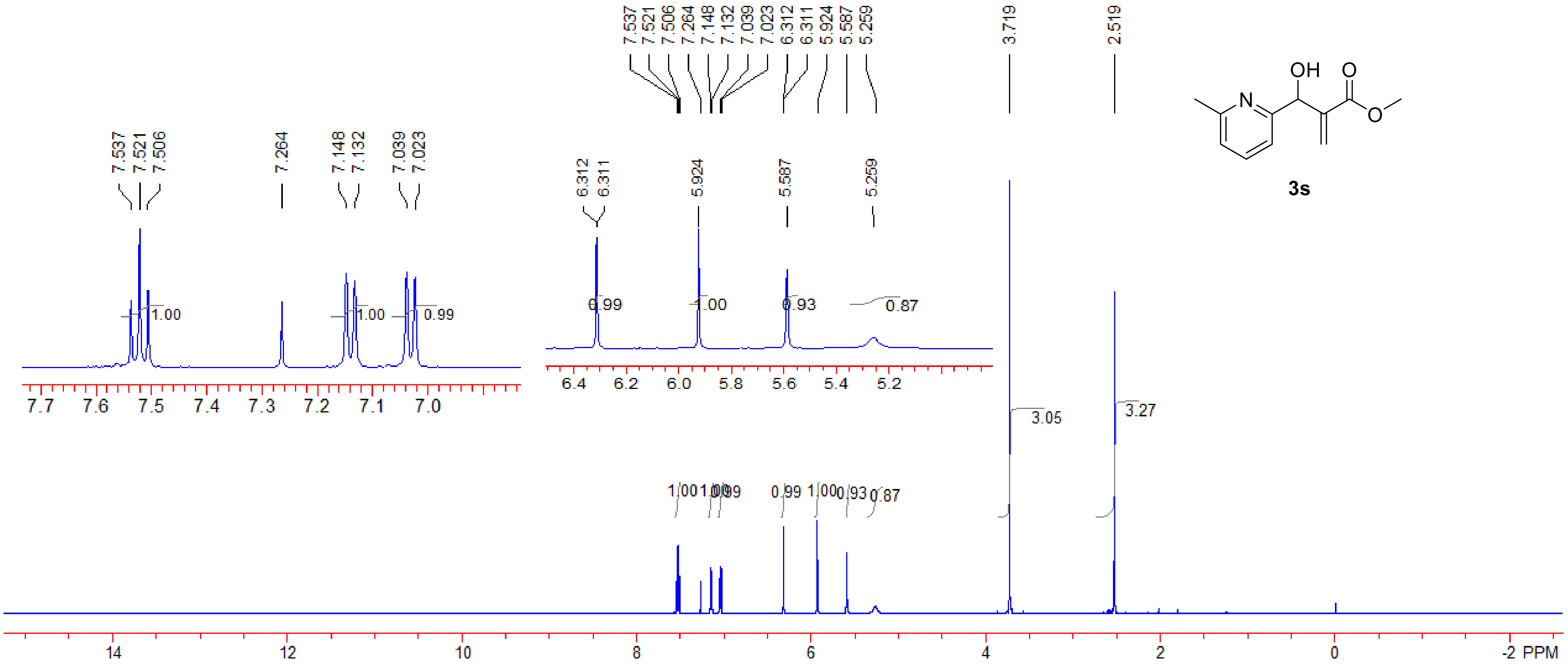

3s, ^{13}C NMR, 126 MHz, $CDCl_3$

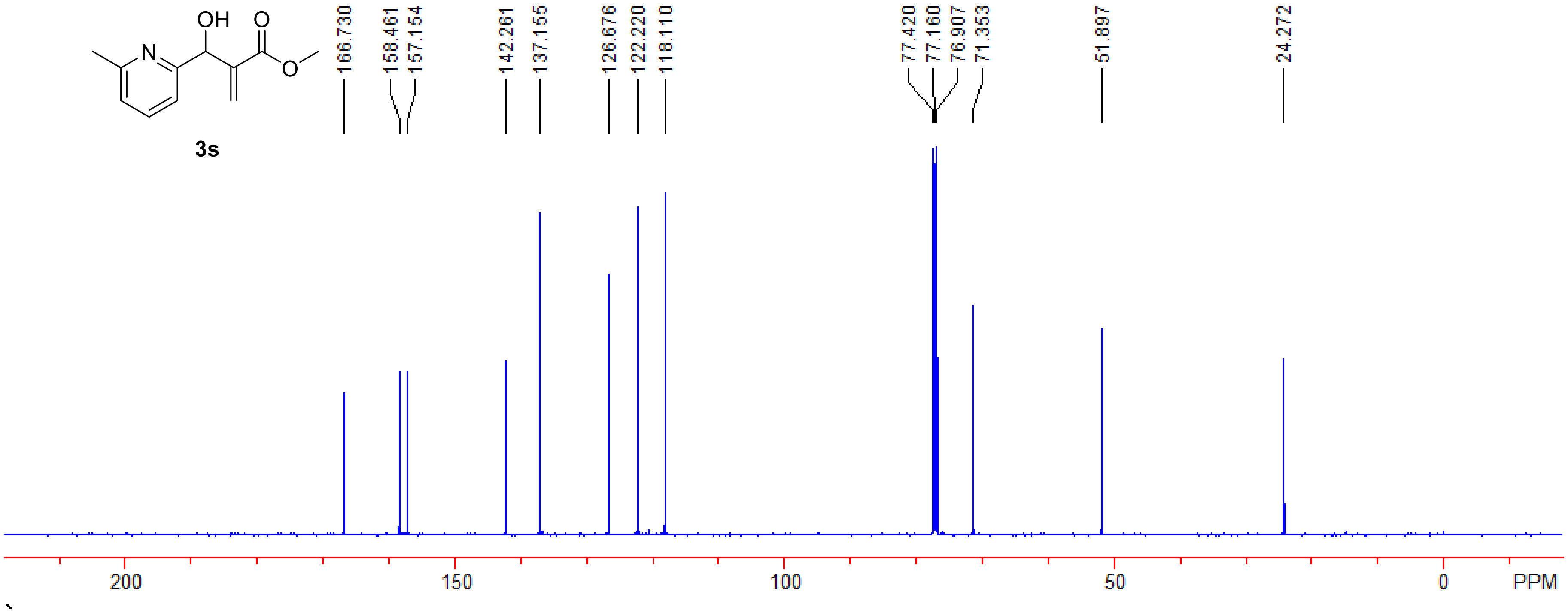

3t, ^{1}H NMR, 500 MHz, $CDCl_3$

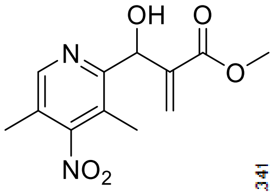

3t

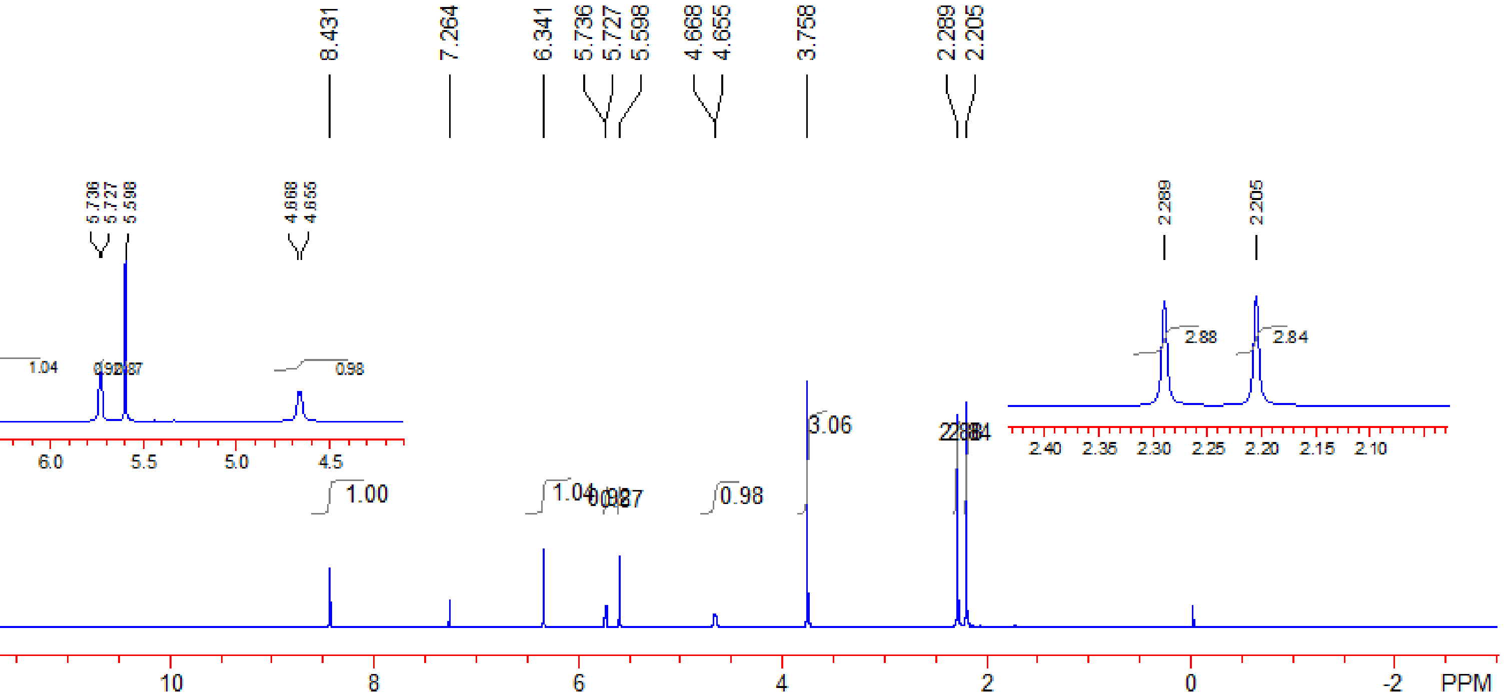

3t, ^{13}C NMR, 126 MHz, $CDCl_3$

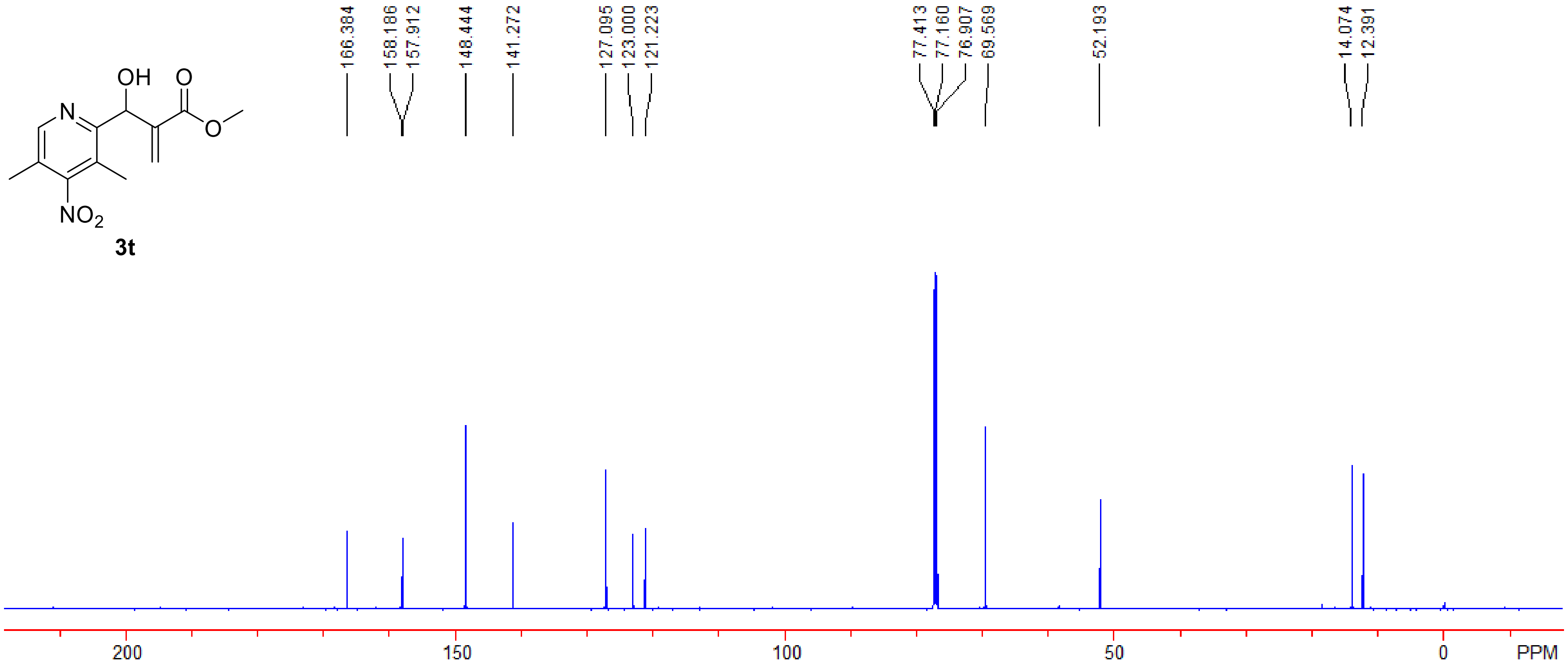

3u, ^{1}H NMR, 500 MHz, $CDCl_3$

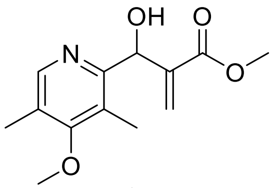

3u

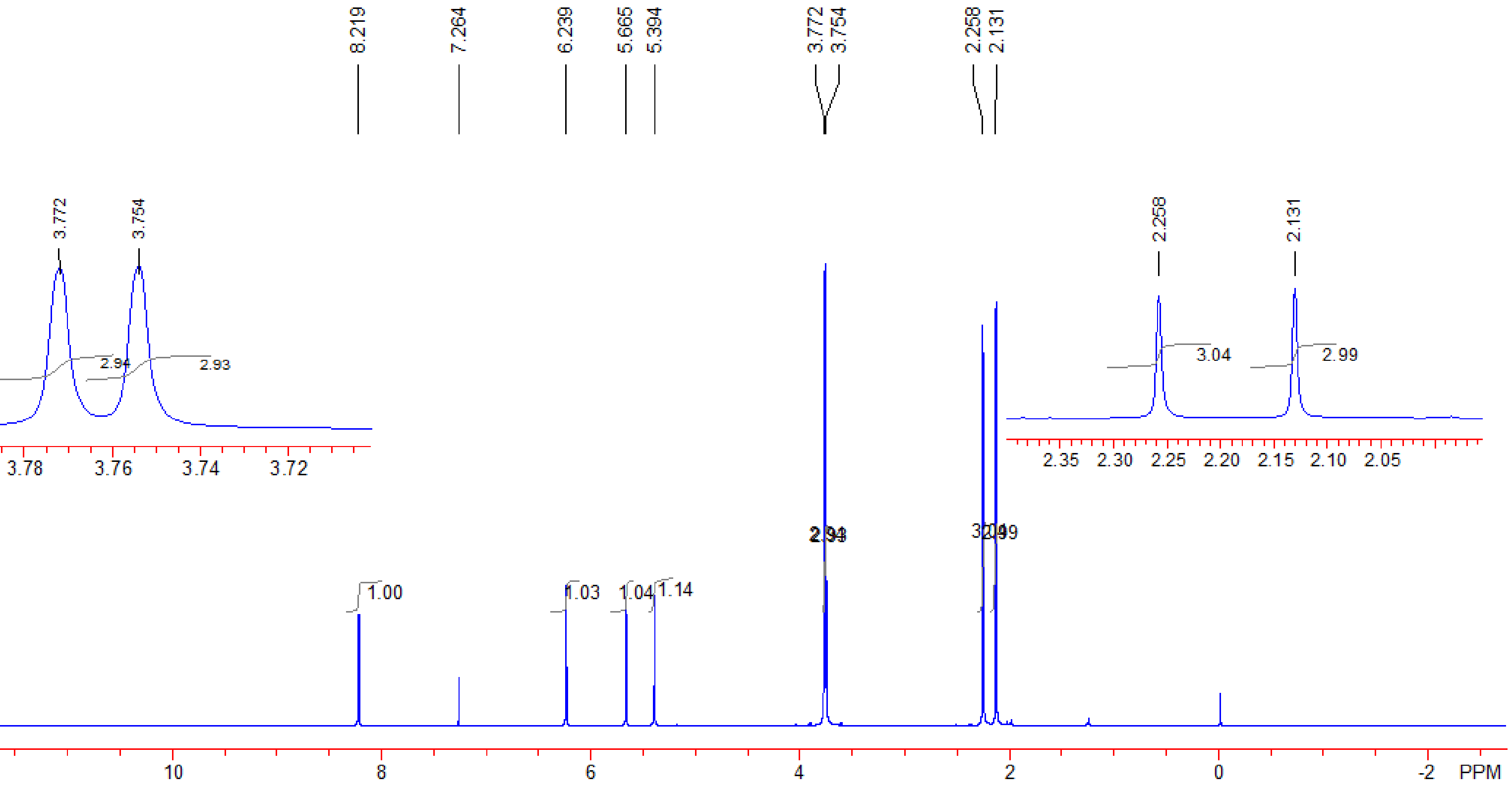

3u, ^{13}C NMR, 126 MHz, $CDCl_3$

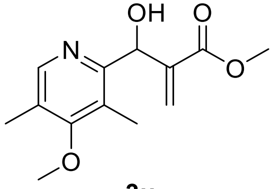

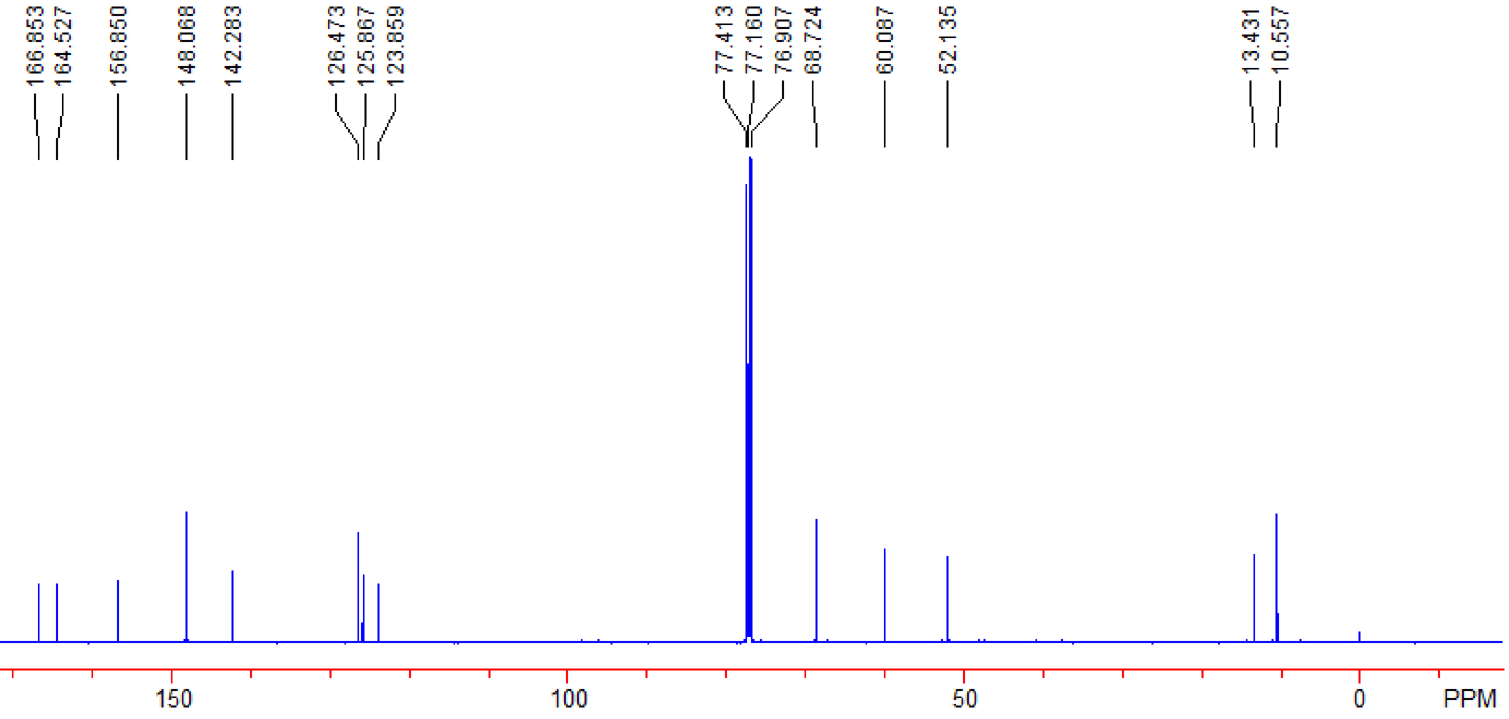

3v, ^{1}H NMR, 500 MHz, $CDCl_3$

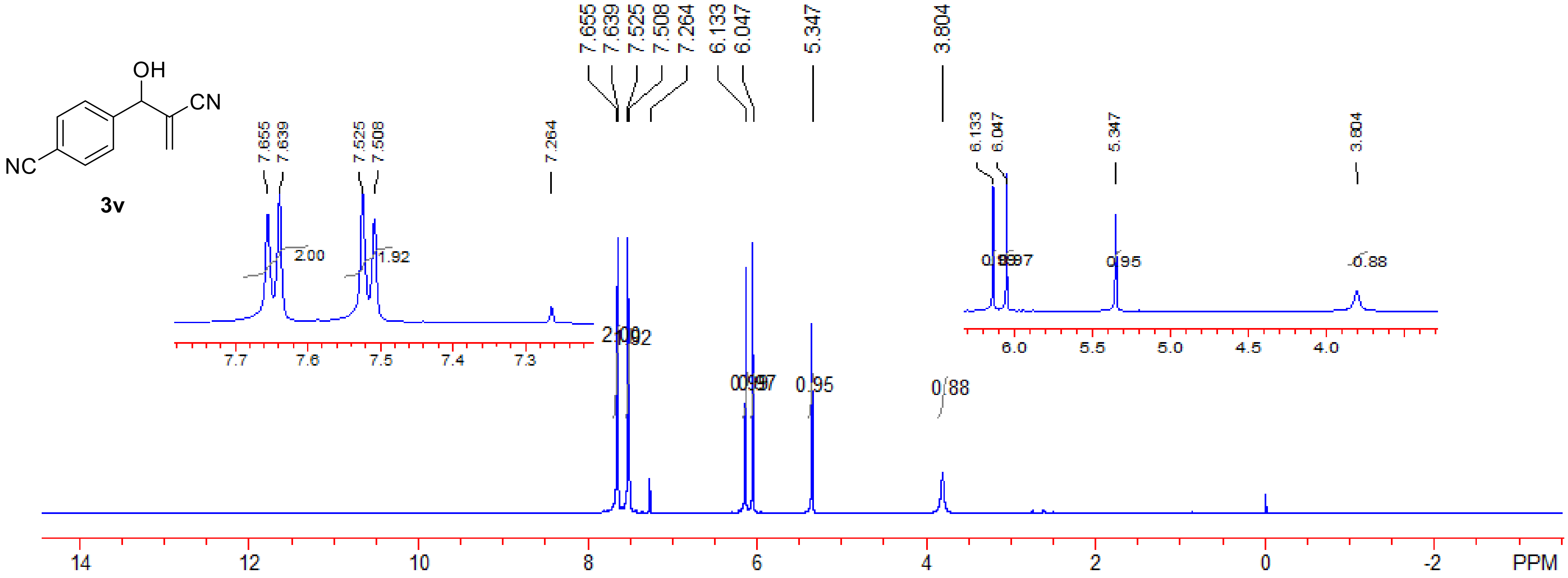

3v, ^{13}C NMR, 126 MHz, $CDCl_3$

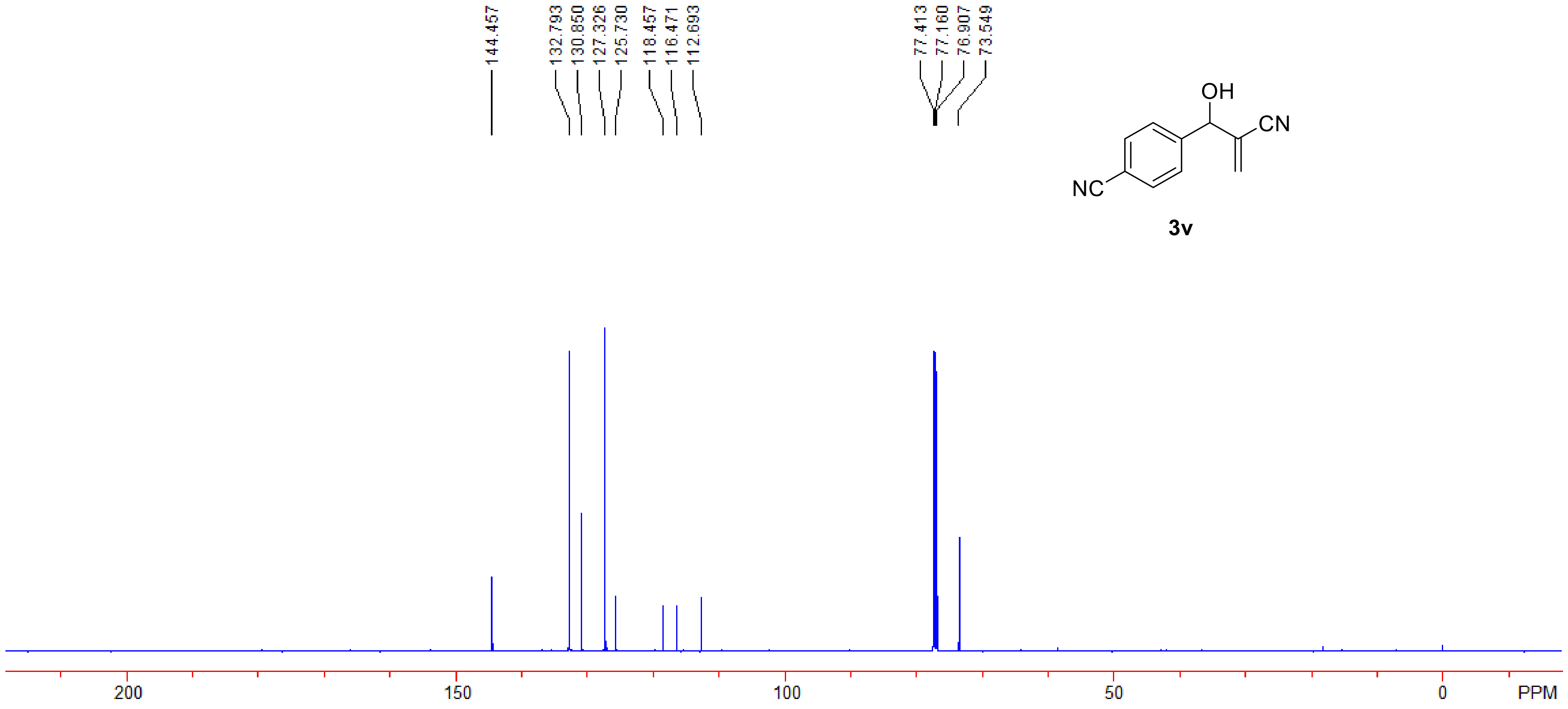

3w, ^{1}H NMR, 500 MHz, $CDCl_3$

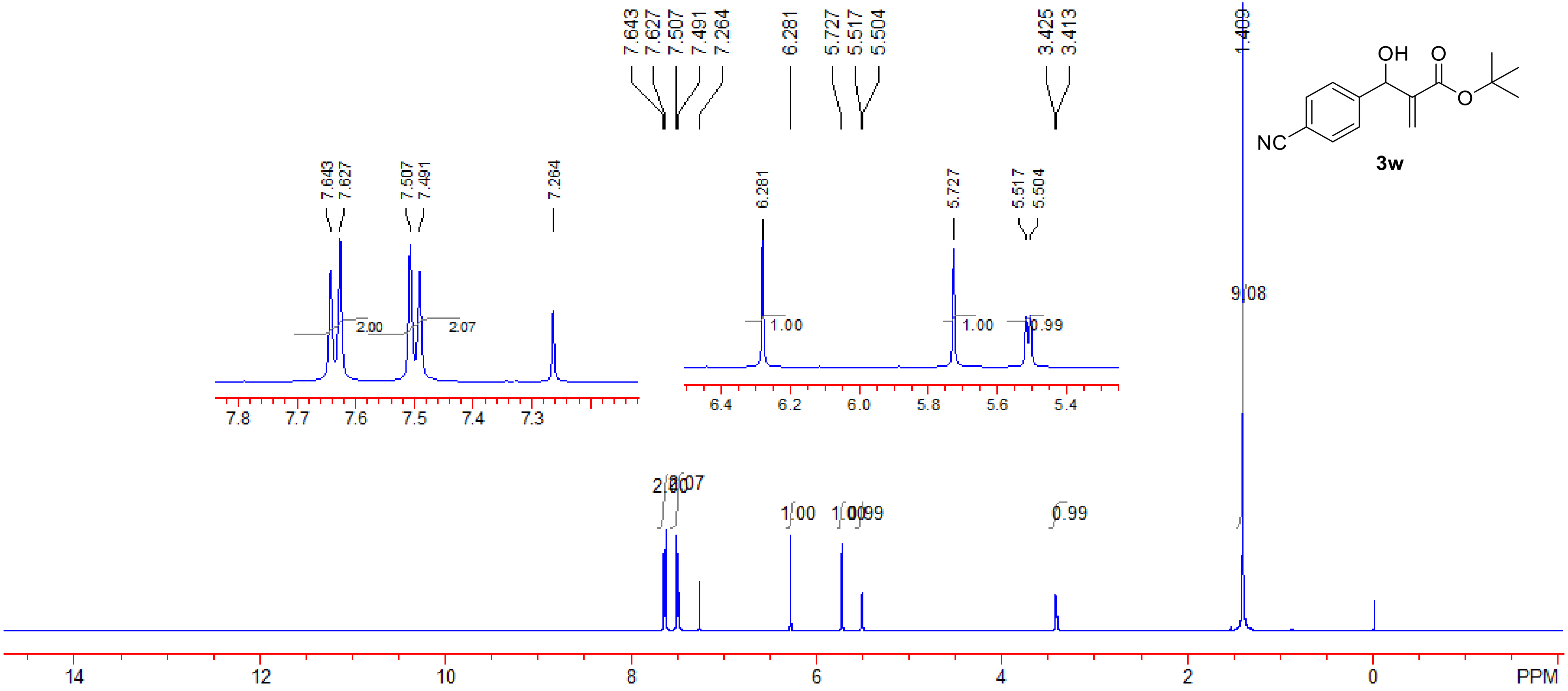

3w, ^{13}C NMR, 126 MHz, $CDCl_3$

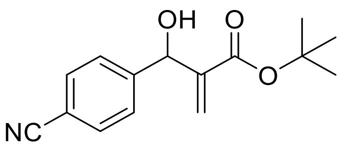

3w

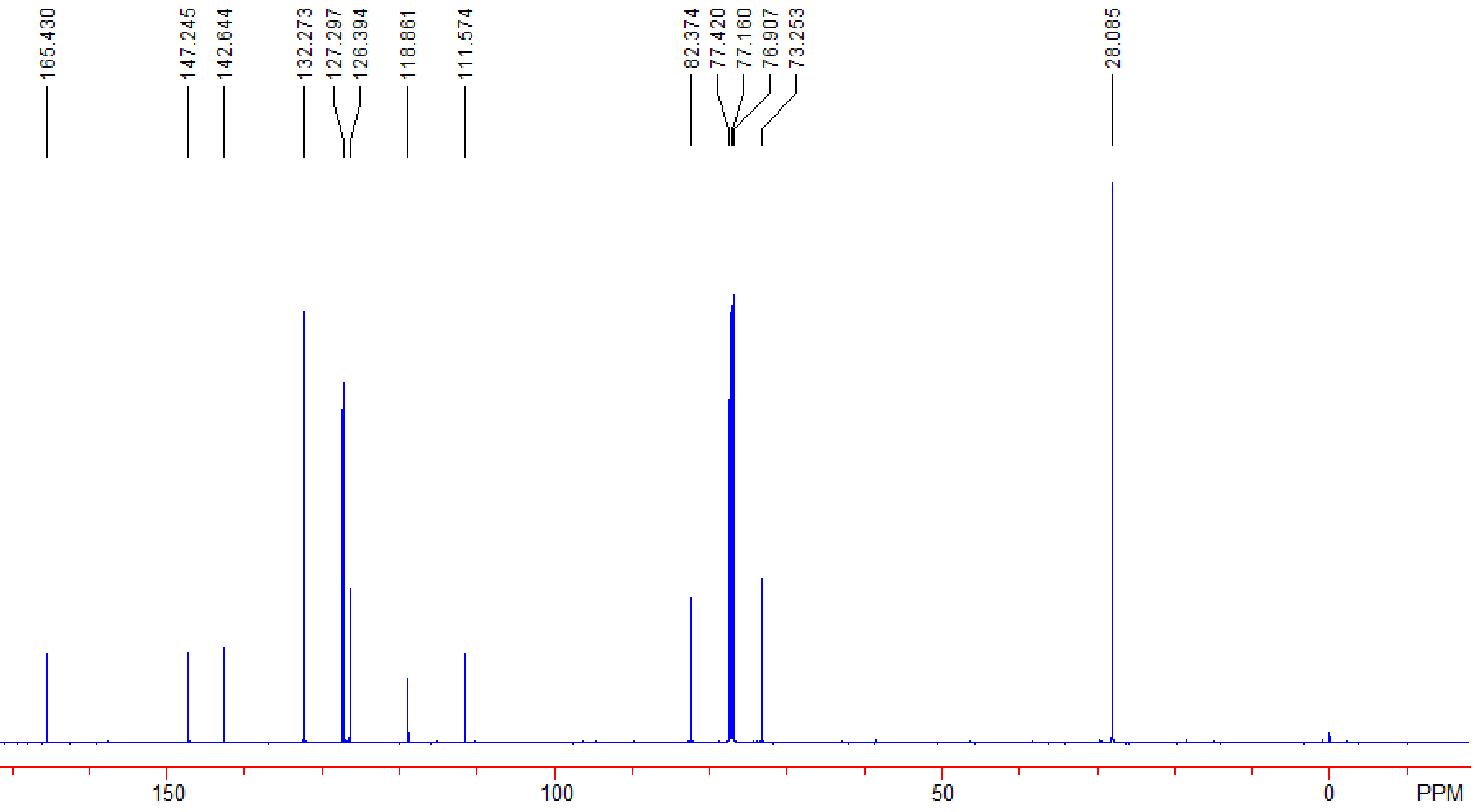

3x, ^{1}H NMR, 500 MHz, CDCl$_3$

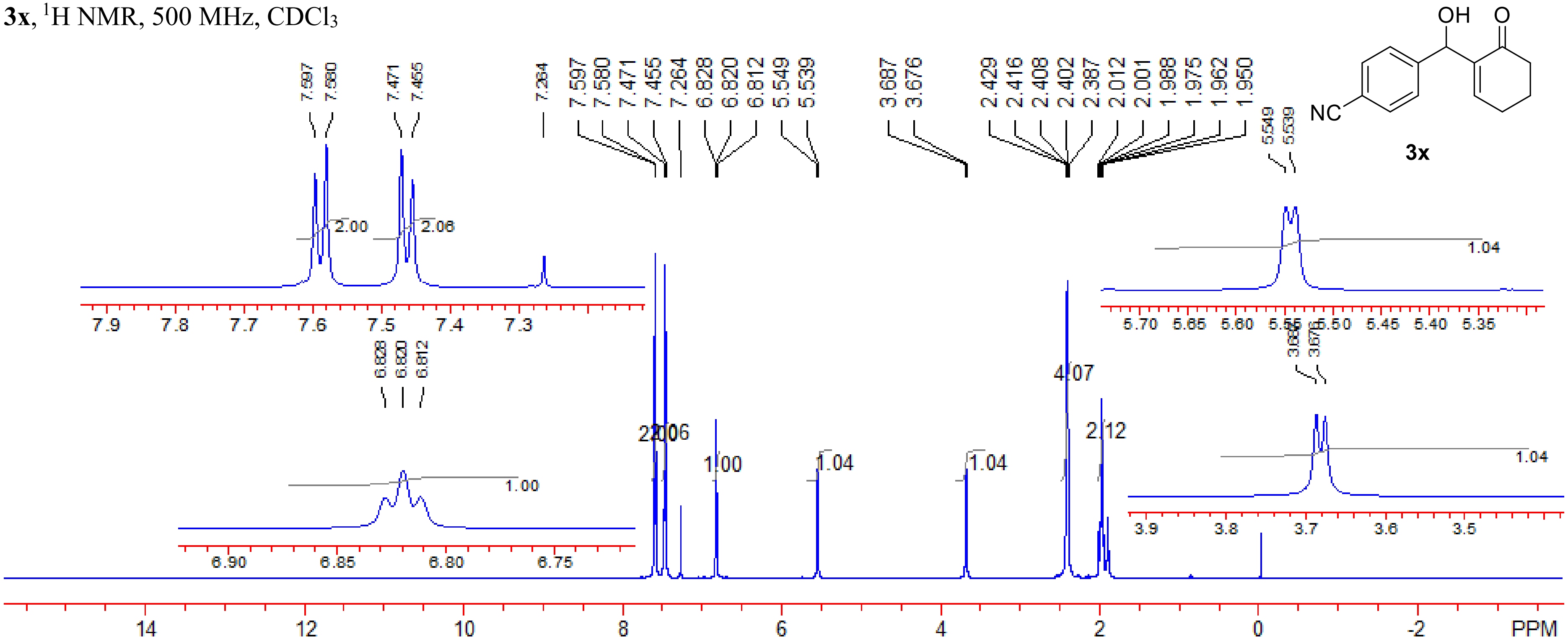

3x, ^{13}C NMR, 126 MHz, CDCl$_3$

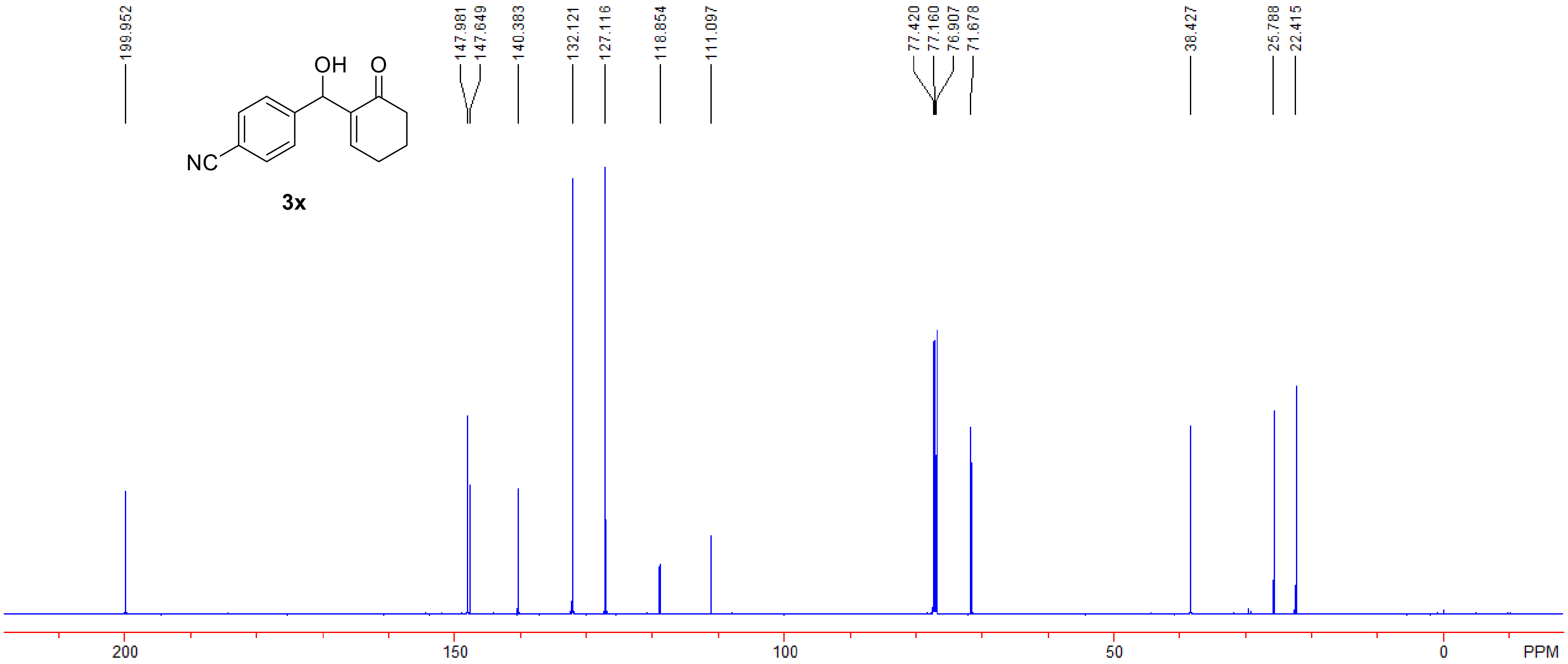

3y, ^{1}H NMR, 500 MHz, CDCl$_3$

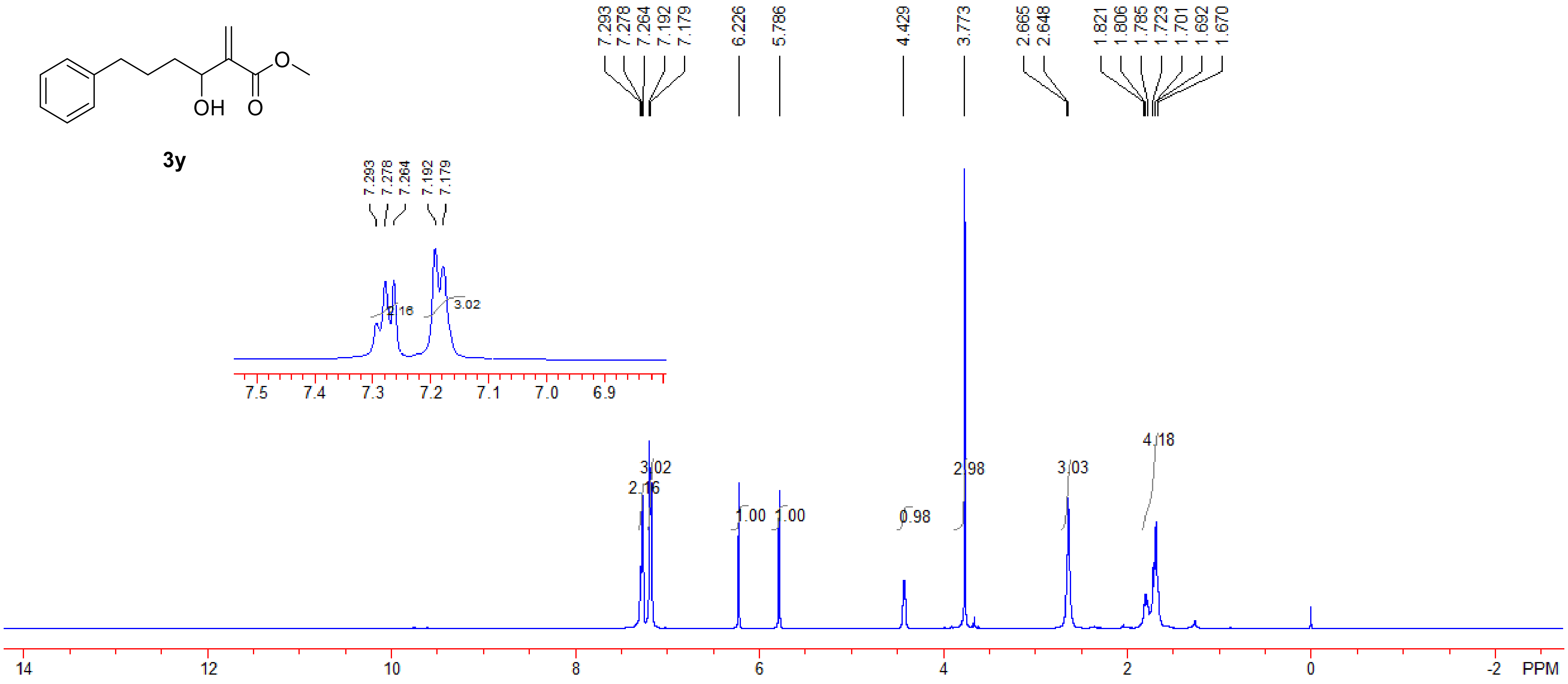

3y, ^{13}C NMR, 126 MHz, $CDCl_3$

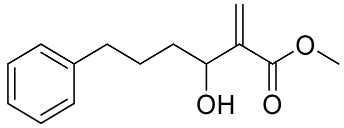

3y

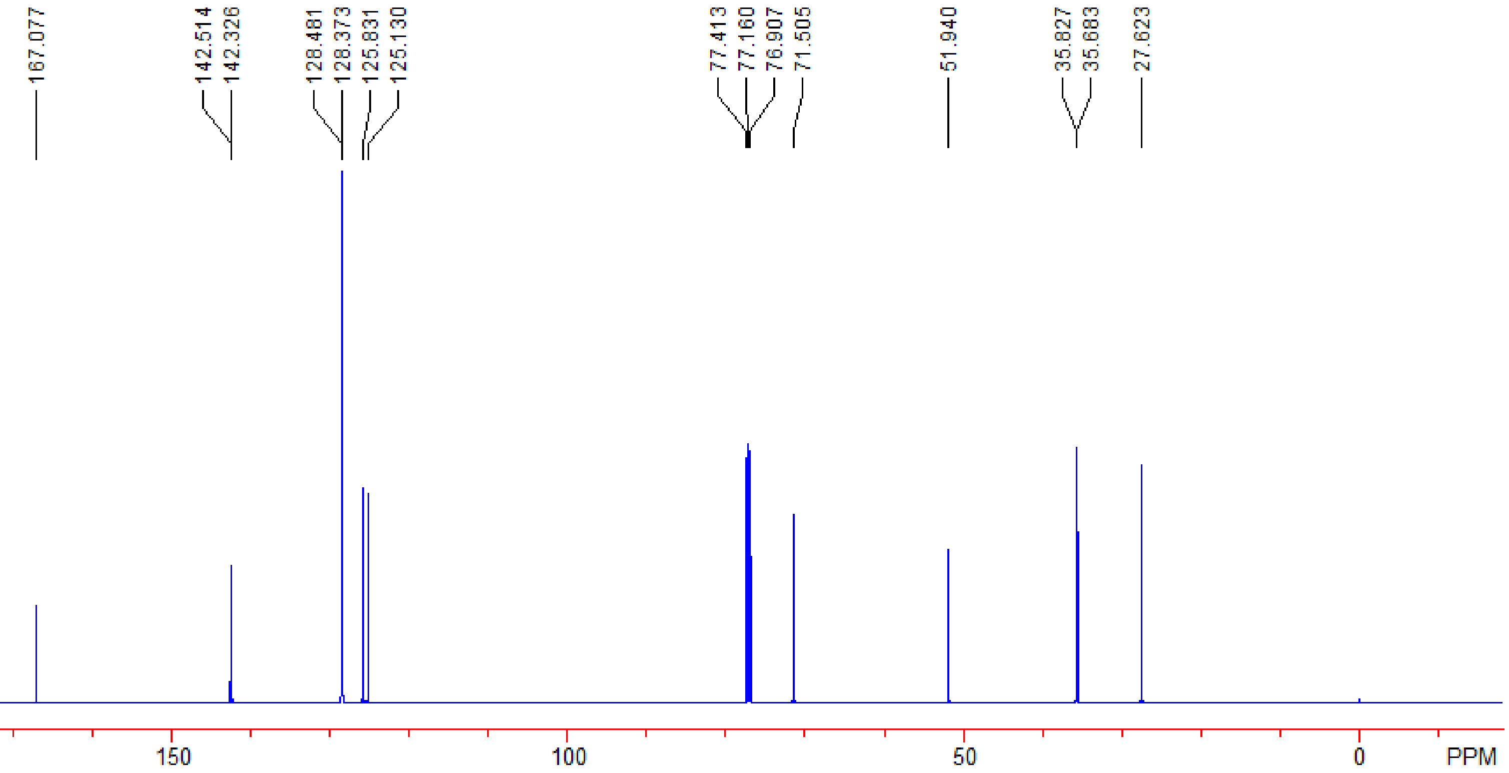

3z, ^{1}H NMR, 500 MHz, $CDCl_3$

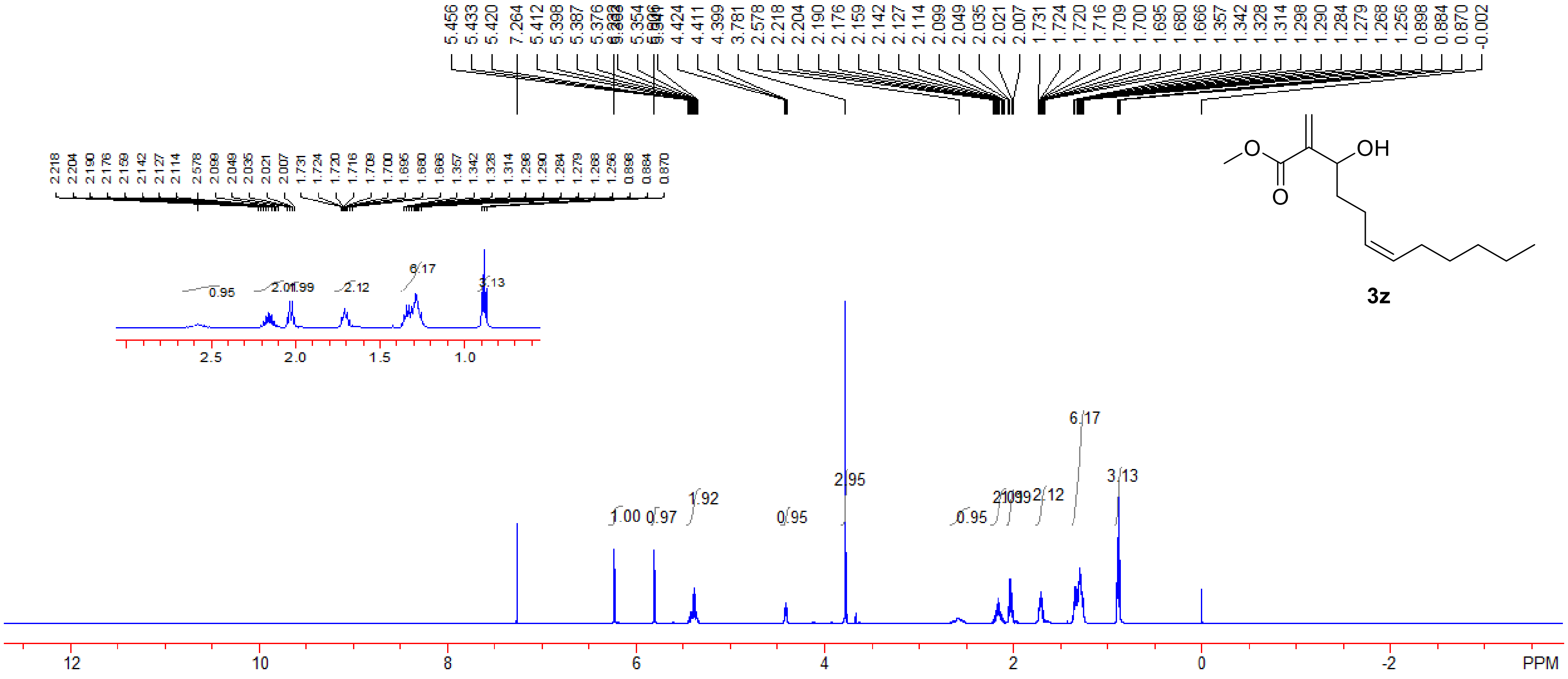

3z

3z, ^{13}C NMR, 126 MHz, $CDCl_3$

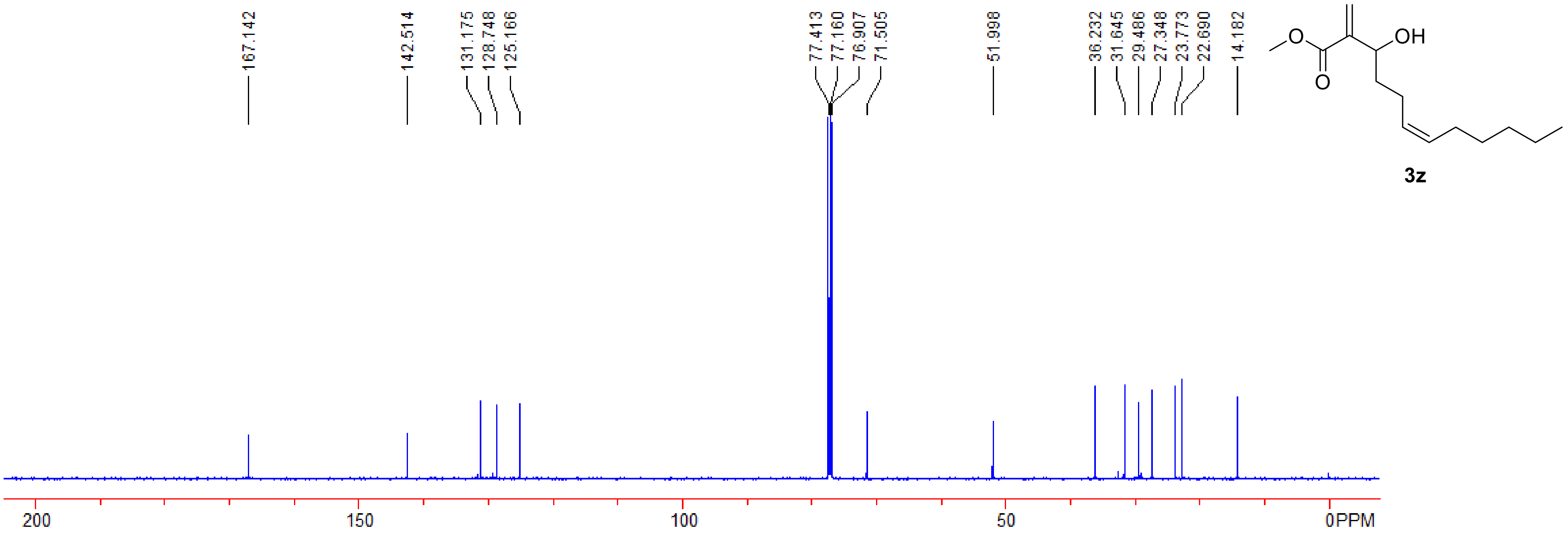

3aa, ^{1}H NMR, 500 MHz, $CDCl_3$

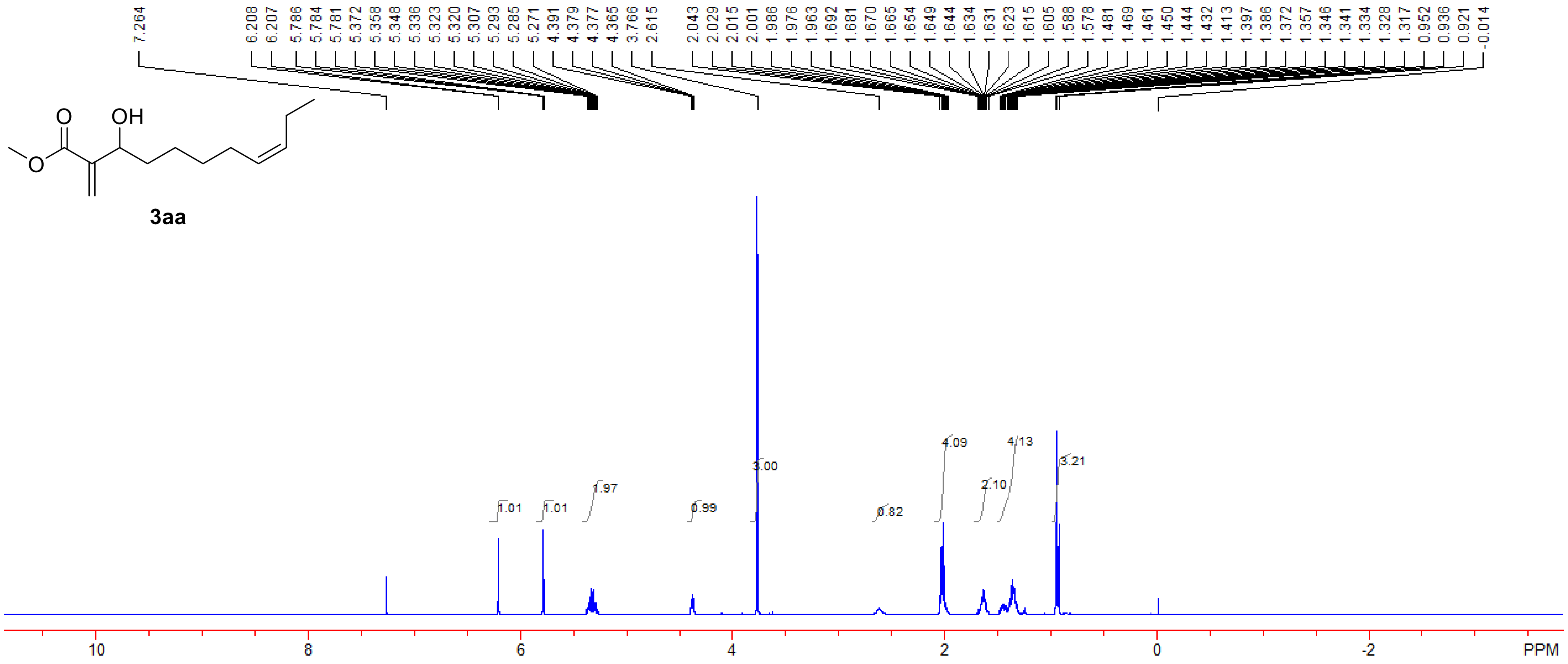

3aa, ^{13}C NMR, 126 MHz, $CDCl_3$

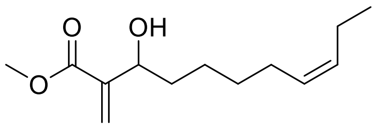

3aa

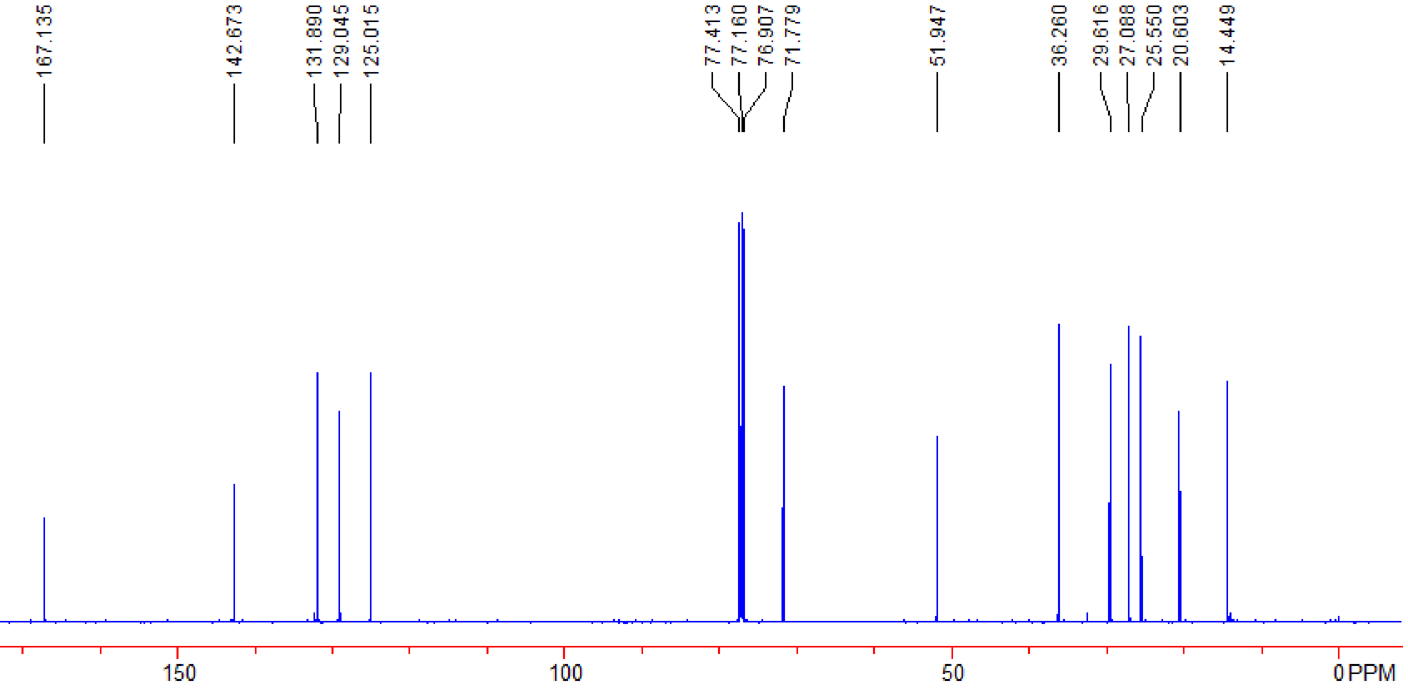

3ab, ^1H NMR, 500 MHz, $CDCl_3$

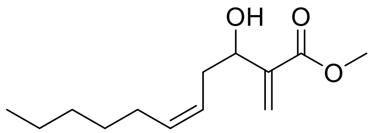

3ab

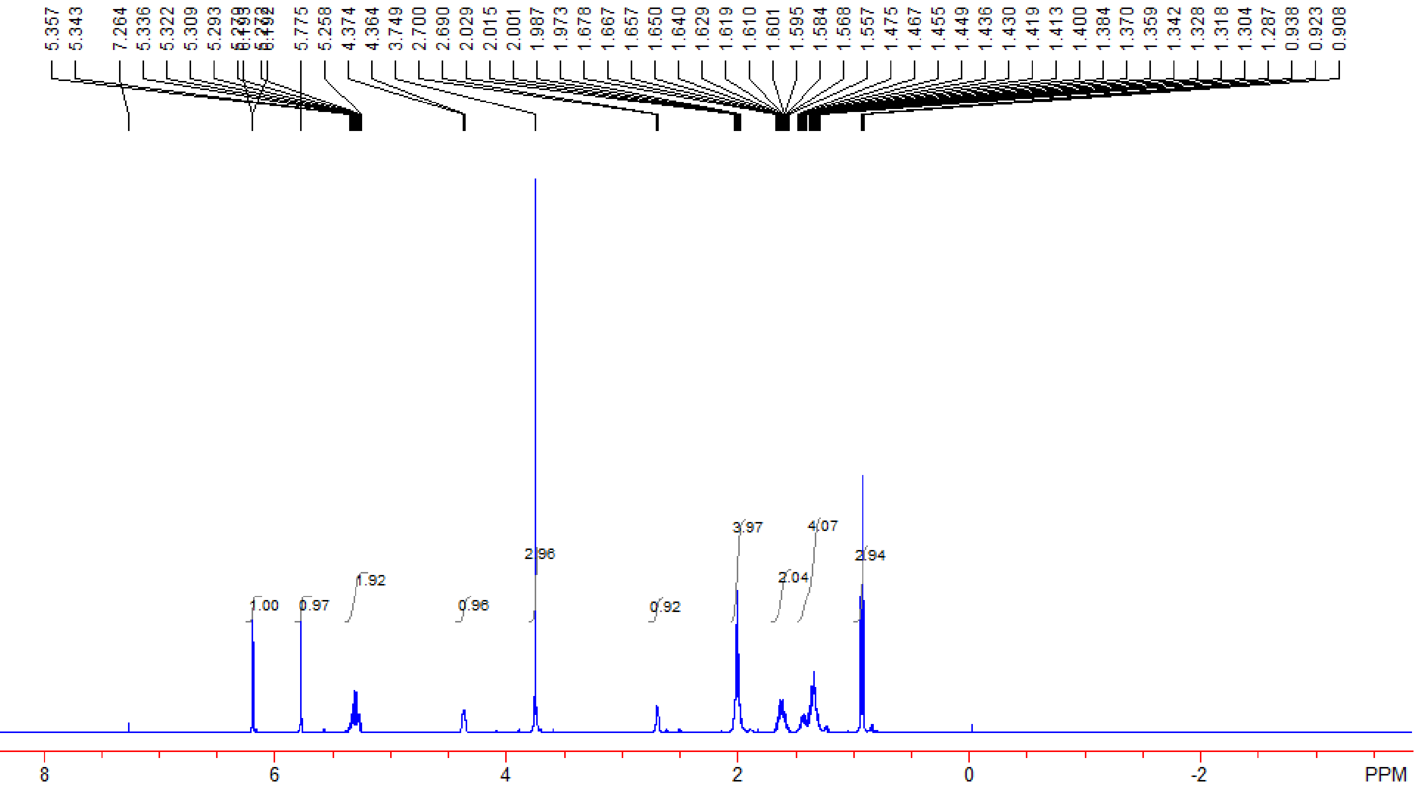

3ab, ^{13}C NMR, 126 MHz, $CDCl_3$

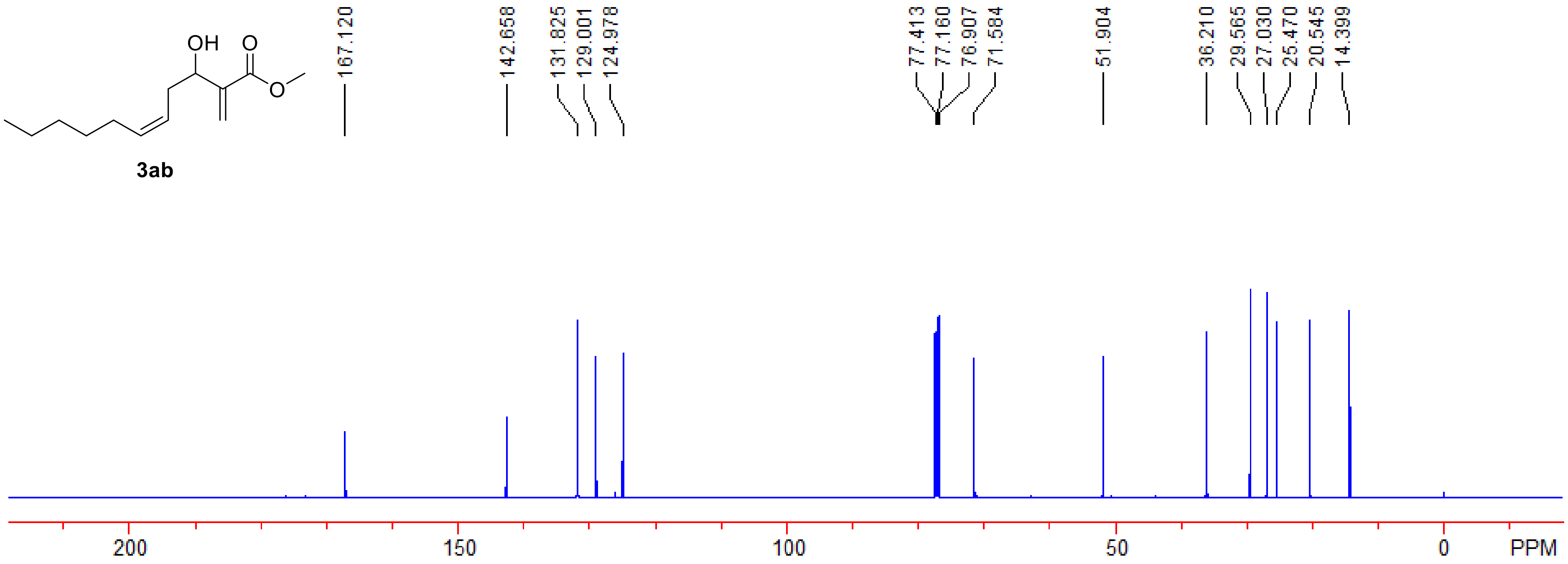

3ab
